# Direct Binding of pRb/E2F-2 to GATA-1 Regulates Maturation and Terminal Cell Division during Erythropoiesis

**DOI:** 10.1371/journal.pbio.1000123

**Published:** 2009-06-09

**Authors:** Zahra Kadri, Ritsuko Shimizu, Osamu Ohneda, Leila Maouche-Chretien, Sylvie Gisselbrecht, Masayuki Yamamoto, Paul-Henri Romeo, Philippe Leboulch, Stany Chretien

**Affiliations:** 1 CEA, Institute of Emerging Diseases and Innovative Therapies, Fontenay-aux-Roses, France; 2 UMR INSERM U.962, University Paris XI, CEA, Fontenay-aux-Roses, France; 3 Département d'Hématologie, INSERM U567, CNRS UMR 8104, Institute Cochin and University Paris V René Descartes, Paris, France; 4 Department of Molecular and Developmental Biology, Center for TARA, ERATO Environmental Response Project, University of Tsukuba, Tsukuba, Japan; 5 Genetics Division, Department of Medicine, Brigham & Women's Hospital and Harvard Medical School, Boston, Massachusetts, United States of America; Baylor College of Medicine, United States of America

## Abstract

How cell proliferation subsides as cells terminally differentiate remains largely enigmatic, although this phenomenon is central to the existence of multicellular organisms. Here, we show that GATA-1, the master transcription factor of erythropoiesis, forms a tricomplex with the retinoblastoma protein (pRb) and E2F-2. This interaction requires a LXCXE motif that is evolutionary conserved among GATA-1 orthologs yet absent from the other GATA family members. GATA-1/pRb/E2F-2 complex formation stalls cell proliferation and steers erythroid precursors towards terminal differentiation. This process can be disrupted in vitro by FOG-1, which displaces pRb/E2F-2 from GATA-1. A GATA-1 mutant unable to bind pRb fails to inhibit cell proliferation and results in mouse embryonic lethality by anemia. These findings clarify the previously suspected cell-autonomous role of pRb during erythropoiesis and may provide a unifying molecular mechanism for several mouse phenotypes and human diseases associated with GATA-1 mutations.

## Abstract 2

Cell differentiation is often coupled with cell cycle arrest. Here, we show that direct binding of the erythroid transcription factor GATA-1 to the retinoblastoma protein and the pRb/E2F transcription factor complex is critical for red blood cell formation.

## Author Summary

Red blood cell production, or erythropoiesis, proceeds by a tight coupling of proliferation and differentiation. The earliest erythroid progenitor identifiable possesses remnant stem cell characteristics as it both self-renews and differentiates. Each progenitor gives rise to more than 10,000 cells, including secondary progenitors. Yet, during the next stage of differentiation, much of this renewal capability is lost, and terminal erythroid differentiation progresses in a stepwise manner through several stages separated by a single mitosis. The transcription factor GATA-1 is essential for erythroid differentiation because it induces the expression of all the known erythroid-specific genes. Here, we show that GATA-1 directly interacts with proteins that are central to the process of cell division: the retinoblastoma protein pRb and the transcription factor E2F. Specifically, E2F becomes inactivate after engaging in a GATA-1/pRb/E2F tricomplex. Another erythroid transcription factor, termed FOG-1, is able to displace pRb/E2F from this complex in vitro upon binding to GATA-1. We hypothesize that the liberated pRb/E2F can then be the target of subsequent regulation to ultimately release free E2F, which triggers cell division. The physiological role of this new pathway is evidenced by transgenic mouse experiments with GATA-1 mutants unable to bind pRb/E2F, which result in embryonic lethality by anemia.

## Introduction

With more than 100 billion red blood cells generated every day, the erythroid lineage has the largest quantitative output of cell production in adult mammals. This impressive capability requires a pattern of cell proliferation closely related to that of embryonic cells followed by ultimate inhibition of cell division, when terminal erythroid differentiation is completed. Yet, the putative molecular pathways that coordinate cell proliferation and erythroid differentiation remain obscure. The transcription factor GATA-1 is essential for erythroid differentiation as it transactivates all the known erythroid-specific genes upon binding to specific DNA motifs [1],[2]. GATA-1 also exerts a repressive action on a subset of genes [3], and its overexpression inhibits cell proliferation [4],[5]. The cofactor Friend-of-GATA-1 (FOG-1) binds to GATA-1 [6] and modulates its activity on given target genes, and mice deficient in either GATA-1 [7],[8] or FOG-1 [9] die from severe anemia. Perturbation of the cell proliferation machinery also commonly results in lethal fetal anemia, as seen in mice defective in pRb [10]–[12], the three cyclins D together [13], more than one E2F members or Cdk4/6 [14],[15]. With respect to the role of pRb in erythropoiesis during development, conflicting views persist as to its cell-autonomous (intrinsic) or nonautonomous (extrinsic) nature, the latter involving the accessory contribution of macrophages [16] or even the placenta [17] as the primary cause for embryonic lethality. Yet, other studies support the existence of a cell-autonomous component for pRb in erythropoiesis, although the underlying molecular pathways remain unknown [18]–[21]. Particularly puzzling is the phenotypic paradox of mutations of the GATA-1 gene that result in the synthesis of an N-terminally truncated GATA-1 protein (GATA-1s) [22]. In a family of patients with an inherited mutation of the GATA-1 gene that results in GATA-1s expression, a severe anemia occurs [23]. In contrast, patients with the Down syndrome (trisomy 21) are prone to cellular selection of acquired somatic GATA-1 mutations that produce GATA-1s and result in preleukemic myeloproliferative disorders [24].

Here, we provide evidence of a direct physical interaction between pRb/E2F-2 and GATA-1 and document its physiological significance. We have also uncovered a potentially novel function for FOG-1 as a regulator of pRb for the control of cell proliferation. This direct interplay between GATA-1, FOG-1, pRb, and E2F sheds a new light on a constellation of mouse phenotypes and human syndromes that implicate mutations of the *Rb* or *GATA-1* genes.

## Results

### Direct Interaction between GATA-1 and pRb Requires the Integrity of a LXCXE Motif

**Figure 1:**
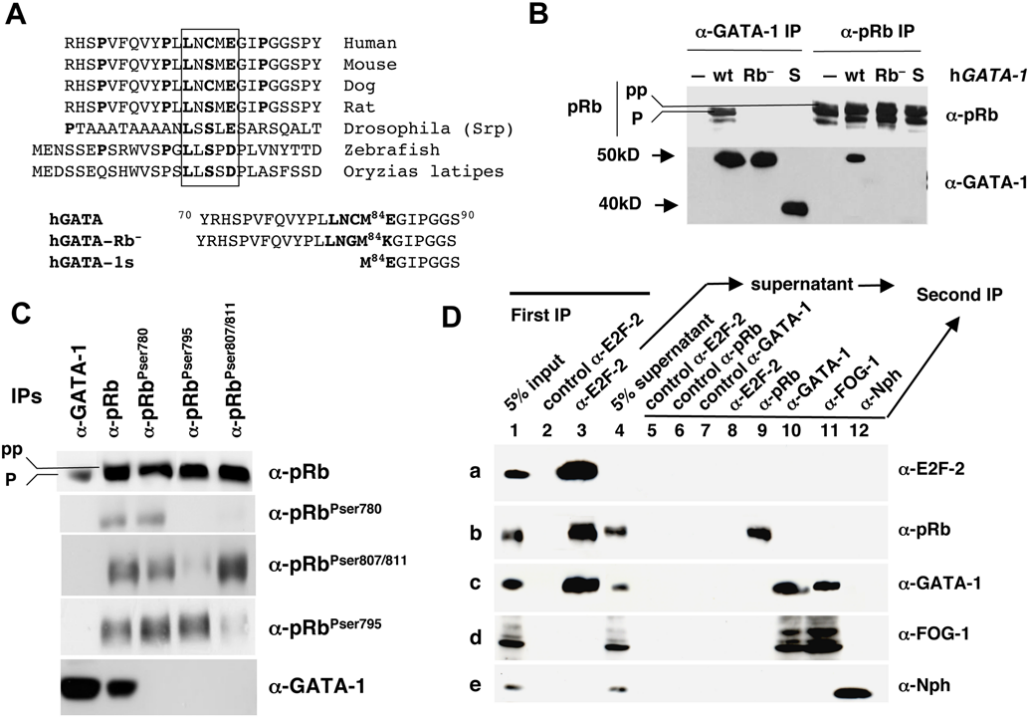
The GATA-1 LXCXE motif mediates the GATA-1/pRb interaction. (A) Top, protein sequence alignment of GATA-1 orthologs. The consensus pRb association motif, LXCXE, is boxed. Bottom, protein sequence alignment of GATA-1 mutants used in Figure 2B. (B) Co-IP assays of nuclear lysates from retrovirally transduced NIH-3T3 cells expressing wt hGATA-1, hGATA-1 bearing two amino acid substitutions that disrupt the LNCME motif (Rb^−^), or hGATA-1 lacking 83 NH_2_ amino acids (S). Corresponding amino acid sequences are indicated at the bottom of Figure 1A. The initial IP step was performed with either anti(α)-GATA-1 (C-20; Santa Cruz Biotechnology), which recognizes both wt and S forms of GATA-1, or anti(α)-pRb (BD Pharmingen) Abs. The immunoprecipitates were subsequently resolved by western blot analysis with either one of the same Abs, i.e., anti(α)-pRb or anti(α)-GATA-1. Hypo (“p”) and hyperphosphorylated (“pp”) pRb forms are indicated. An identical experiment, demonstrating that signals were not immunoglobulin artifacts (IgG isotype species-specific controls for all the Abs used), is shown in Figure S1B. (C) Co-IPs of nuclear extracts from the erythroid cell line UT-7 were performed with either (α)-GATA-1 (C20; Santa Cruz Biotechnology) or (α)-pRb (BD Pharmingen) Abs. Four different anti-pRb Abs were used with different specificities: α-pRb (BD Pharmingen) recognizes all forms of pRb regardless of their phosphorylation status; the other three Abs recognize pRb phosphorylated on either Ser^780^, Ser^795^, or Ser^807/811^ (Cell Signaling Technology). The precipitated proteins were subsequently analyzed by western blot using the same pRb Abs or an α-GATA-1 Ab (N1; Santa Cruz Biotechnology). (D) A first IP of nuclear extracts from purified CD71^+^ (>90 TER119^+^) late-stage erythroid cells from E12.5 mouse fetal liver was performed with an α-E2F-2 Ab. The first IP immunoprecipitate was analyzed in lane 3, with its IgG species-specific isotype control (lane 2). The E2F-2 immunodepleted supernatant was then submitted to secondary IPs with either α-E2F-2 (lane 8), α-pRb (lane 9), α-GATA-1 (lane 10), α-FOG-1 (lane 11), or the negative control α-Nucleophosmin (Nph) (lane 12) Abs. The relevant IgG species-specific isotype controls are shown in lanes 5, 6, and 7 (see experimental procedure in Figure S2E). Initial 5% input and 5% supernatant controls were analyzed in lanes 1 and 4, respectively. Primary (α-E2F-2) IP, secondary IPs, and the aforementioned controls were subsequently resolved by western blot analysis with the indicated Abs (rows a to e).

By examining the coding sequence of human GATA-1 (hGATA-1), we have identified an LNCME motif located at amino acid positions 81 to 85. This sequence exactly matches the consensus LXCXE motif present in many cellular or viral pRb-binding proteins [25],[26]. Peptidic alignment of all known GATA-1 orthologs shows the presence of the LXCXE or its variant LXSXE in all species (Figure 1A). Serine is structurally similar to cysteine but contains a hydroxyl (-OH) group in place of the thiol (-SH) group. Neither of these two motifs (LXCXE and LXSXE) is present in any of the other members of the GATA family (i.e., GATA-2 to -6; unpublished data). To probe for the functionality of this putative Rb-binding domain in hGATA-1 in vivo, NIH-3T3 cells, which do not express GATA-1, were transduced with retroviral vectors that encode either mGATA-1 or hGATA1. Cross coimmunoprecipitation (co-IP) analysis with GATA-1 and pRb (p110) specific antibodies (Abs) confirmed the hypothesis that pRb is associated with h/mGATA-1 in this reconstituted model (Figure S1A), thus suggesting that both LXCXE and LXSXE motifs are functional within the GATA-1 proteins for pRb binding. To demonstrate that pRb/hGATA-1 interaction is specifically dependent upon the LNCME motif, NIH-3T3 cells were transduced with retroviral vectors that express either (1) hGATA-1, (2) a naturally occurring N-terminally truncated form of hGATA-1, referred to as hGATA-1s (“s” for “short” [24]), which is initiated at methionine 84 within the LNCM^84^E motif, or (3) hGATA-1 bearing two amino acid substitutions within the LNCME motif (LNCME to LNGMK; referred to as hGATA-1Rb^−^) (Figure 1A). Cross co-IP with GATA-1– and pRb-specific Abs showed that only wild-type (wt) hGATA-1 interacts with pRb, whereas hGATA-1Rb^−^ and hGATA-1s do not (Figure 1B), thus establishing that the LNCME motif in hGATA-1 is required for direct association of pRb to GATA-1. In all the aforementioned co-IP experiments, potential immunoglobulin (Ig) artifacts were ruled out by analyzing IgG isotype controls for all the Abs used and for each GATA-1 variant expressed, under the same experimental conditions (Figure S1B).

### Molecular Composition of the GATA-1/pRb Complex: Presence of E2F-2 and Absence of FOG-1

Because pRb activity is regulated by phosphorylation, we analyzed the pRb phosphorylation status within the GATA-1/pRb complex in erythroid (UT7) and nonerythroid (NIH3T3) cells. There are 16 possible CDK phosphorylation sites (Ser/Thr-Pro motifs) in pRB. By western blot analysis, Rb proteins segregate into two distinctly clustered groups of migrating bands: the more slowly migrating correspond to the so-called “hyperphosphorylated” forms, whereas the faster migrating are the “hypophosphorylated” forms, without defined species phosphorylated on specific residues among the 16 always present. Co-IP with an anti–GATA-1 Ab followed by western blot analysis with an anti-pRb Ab indicated that only the hypophosphorylated (“p”) forms of pRb were involved in the GATA-1/pRb complex, while one could detect both hypophosphorylated and (“pp”) hyperphosphorylated forms of pRb by IP of the original sample with an anti-pRb antibody (Figure 1B). Because available pRb antibodies are notoriously fastidious at differentiating hypo- from hyperphosphorylated pRb with optimal clarity, an additional experiment was performed by means of antibodies to phospho-pRb. Co-IP was first performed with specific phospho-pRb antibodies (Pser^780^, Pser^807/811^, or Pser^795^) before resolution by western blot analysis reacted with a GATA-1 antibody. We found that GATA-1 was not precipitated under these conditions (Figure 1C).

Because hypophosphorylated pRb is known to bind members of the E2F family and actively suppress their transcriptional activity, whereas it has been recently reported that only E2F-2—among all the E2F members present in end-stage (CD71^+^, TER119^+^) fetal liver erythroid cells—directly interacts with pRb (p110) [20], we investigated whether E2F-2 is present within the GATA-1/pRb complex. To this end, we purified CD71^+^ erythroid cells (>90% TER119^+^) from embryonic day (E)12.5 mouse fetal liver. We first established that all Abs used were efficient and specific by immunoprecipitation (IP) and western blot analysis and that signals were not Ig artifacts (IgG isotype controls), especially for GATA-1 proteins that migrate at levels similar to heavy Ig chains (50 kDa) (Figure S2B). Results of co-IP experiments demonstrated that GATA-1, pRb, and E2F-2 do exist as a tricomplex in nuclear extracts from purified E12.5 mouse fetal liver erythroid cells (Figure S2C and S2D).

To further characterize the molecular composition of the GATA-1/pRb/E2F-2 tricomplex, we performed a co-IP assay with an Ab specific for E2F-2 followed by western blot analysis reacted with Abs specific for either pRb, GATA-1, or FOG-1, the known partner of GATA-1. Whereas GATA-1 and pRb proteins could be identified within the E2F-2 immunoprecipitate, FOG-1 was not found (Figure 1D). As we previously demonstrated that GATA-1 is directly associated with pRb and that GATA-1 is not known to bind directly to E2F-2, we can thus surmise that two pRb complexes can possibly coexist in nuclear extracts from fetal liver erythroid cells: pRb/E2F-2 and GATA-1/pRb/E2F-2. To probe whether the presence of E2F-2 is required for GATA-1/pRb complex formation, we first performed an immunodepletion of E2F-2 followed by western blot analysis of the unbound fraction with GATA-1 or pRb specific Abs (Figure S2E). Results indicate that both GATA-1 and pRb could be detected in the unbound fraction (Figure 1D and S2D). The E2F-2 immunodepleted fraction was then submitted to secondary co-IPs with Abs specific for either pRb, GATA-1, or FOG-1. Secondary IP precipitates were resolved by western blot analysis with Abs specific for either E2F-2, pRb, GATA-1 or FOG-1. An association between pRb and GATA-1 could not be detected in the E2F-2 unbound fraction (Figure 1D).

From these series of experiments, we can conclude that (1) a GATA-1/pRb/E2F-2 tricomplex, in which pRb is hypophosphorylated, is present in late-stage erythroid cells from mouse fetal livers, (2) the GATA-1 LXCXE motif is required for direct association of pRb/E2F-2 to GATA-1, (3) GATA-1 and pRb do not form a bicomplex in the absence of E2F-2, (4) only E2F-2, but no other form of E2F present in these erythroid cells, allows measurable GATA-1/pRb/E2F tricomplex formation, and (5) FOG-1 is excluded from the GATA-1/pRb/E2F-2 tricomplex.

### FOG-1 Interferes with the GATA-1/pRb/E2F-2 Association and Displaces pRb from GATA-1 by Direct Protein–Protein Interaction

**Figure 2:**
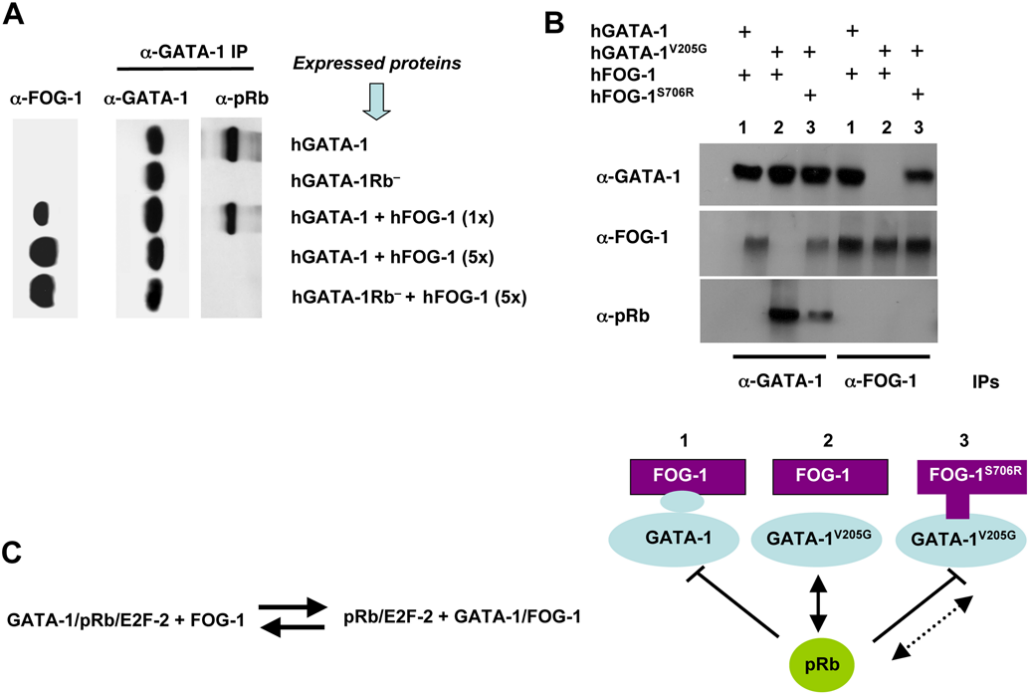
FOG-1 interferes with GATA-1/pRb association. (A) Co-IPs, by means of an anti(α)-GATA-1 Ab, of nuclear extracts from retrovirally transduced NIH-3T3 cells expressing hGATA-1 or hGATA-1Rb^−^ and from the same cells after subsequent transient transfection with increasing amounts (1- to 5-fold, with 1-fold = 0.1 µg) of a hFOG-1 expression plasmid. We verified that >90% cells were successfully transduced by each of the retroviral vectors on the basis of coexpression of eGFP from an internal ribosome entry site (IRES). Immunoprecipitates were then resolved by western blot analysis with either anti(α)-GATA-1 or anti(α)-pRb antibodies, under identical experimental conditions as for Figure 1B and S1. The amount of hFOG-1 expressed after transient transfection was assessed in parallel on total nuclear extracts with an anti(α)-FOG-1 Ab (left column). (B) Nuclear extracts were made from retrovirally transduced NIH-3T3 cells expressing hGATA-1, or hGATA-1^V205G^, which cannot interact with hFOG-1, or 3 d after transient transfection with a plasmid expressing hFOG-1 or an identical amount of an expression plasmid encoding hFOG-1^S706R^, which can interact with hGATA-1^V205G^. We verified that >90% cells were successfully transduced by each of the retroviral vectors on the basis of coexpression of eGFP from an IRES. Co-IP assays were performed with these nuclear extracts by means of α-GATA-1 and α-FOG-1 Abs, followed by western blot analysis with either α-GATA-1, α-FOG-1, or α-pRb Abs. A schematic representation of the various combinations of FOG and GATA variants expressed (lines 1 to 3) is indicated at the bottom of the figure. (C) A proposed protein equilibrium model.

Before addressing the physiological relevance of these findings, we first probed the apparent paradox that FOG-1 is known to be a key cofactor of GATA-1 in erythroid cells, whereas we have now established that FOG-1 is excluded from the GATA-1/pRb/E2F-2 tricomplex. Although the amino acid residues of GATA-1 known to interact with FOG-1 lie within a zinc finger domain located outside the LNCME motif [27],[28], we investigated whether FOG-1 could, nevertheless, regulate the formation of the tricomplex. To this end, we generated pure populations of NIH3T3 cells that express hGATA-1 after retroviral transfer, in which we subsequently transiently transfected various amounts of a plasmid that expresses human FOG-1 (hFOG-1). We chose NIH3T3 cells because they constitutively express E2F proteins, including E2F-2, although they do not naturally express GATA-1 or FOG-1 in the absence of transfection. Co-IP assays showed that hFOG-1 was able, in a dose-dependant manner, to prevent hGATA-1 from forming a complex with pRb (Figure 2A). To assess whether a direct protein–protein interaction between GATA-1 and FOG-1 is required to dissociate pRb from GATA-1, we expressed relevant mutants of hGATA-1 and hFOG-1 in NIH3T3 cells following an identical experimental approach. We made use of the hGATA-1^V205G^ mutant, which comprises a point mutation that substitutes a glycine for a valine at codon 205. This mutation, naturally found in patients and previously studied in mice, is associated with severe dyserythropoietic anemia and thrombocytopenia [28],[29]. It was previously established that this mutation causes the disruption of GATA-1 binding to FOG-1 and, as a consequence, a failure of expression of genes dependent on GATA-1 for their transactivation as well as the lack of repression of the *c-Myc* and *GATA-2* genes [30]. We thus expressed hGATA-1, hGATA-1Rb^−^, or hGATA-1^V205G^ in NIH-3T3 cells by retroviral transfer followed by transient transfection of a plasmid that expresses hFOG-1. Co-IP revealed that hFOG-1 was unable to dissociate hGATA-1^V205G^ from pRb in contrast to hGATA-1 (Figure 2B). As a corroborating demonstration, we used a compensatory hFOG-1 mutant (hFOG-1^S706R^) that is able to bind to hGATA-1^V205G^ and rescue the erythroid maturation of GATA-1^−/−^ cells expressing hGATA-1^V205G^ [30]. Co-IP assays showed that hFOG-1^S706R^ was able to trigger a partial dissociation of hGATA-1^V205G^ from pRb (Figure 2B). The residual association of hGATA-1^V205G^ to pRb in the presence of hFOG-1^S706R^ is likely to result from the lower affinity of hFOG-1^S706R^ for hGATA-1^V205G^ compared to that of hFOG-1 for hGATA-1 (Figure 2B and [30]).

Altogether, we thus conclude that FOG-1 is capable of displacing pRb/E2F from GATA-1 in vitro by direct GATA-1/FOG-1 interaction. An equilibrium model between GATA-1, pRb, E2F-2, and FOG-1 is proposed (Figure 2C).

### Cell Proliferation Is Controlled by Differential Association of GATA-1 to FOG-1 or pRb

**Figure 3:**
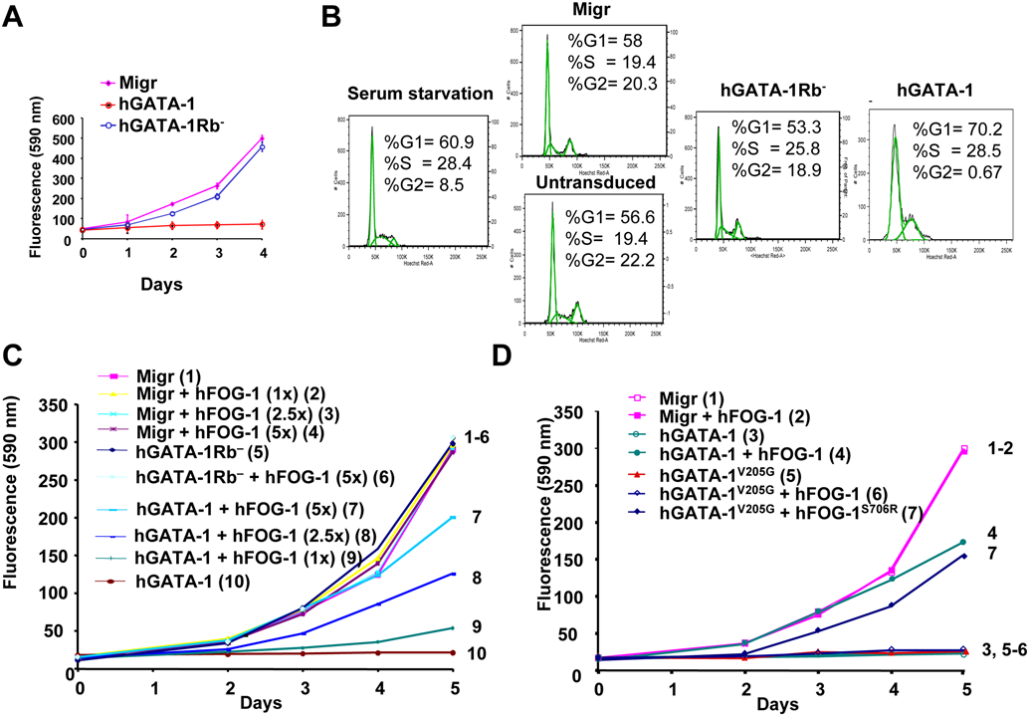
GATA-1/pRb interaction is required for GATA-1–mediated G1/S phase arrest of cell proliferation, which is antagonized by FOG-1 in vitro. (A) Retroviral vectors encoding wt hGATA-1 or the hGATA-1 mutant that cannot interact with pRb (hGATA-1Rb^−^) were used to transduce NIH-3T3 cells, and proliferation of the eGFP-positive NIH-3T3 cells was monitored each day for 4 d by means of the fluorimetric metabolic growth indicator Uptiblue (Interchim) (*n* = 6). Migr indicates NIH3T3 cells transduced with the “empty” retroviral vector (Migr vector), which only expresses eGFP. We verified that >90% cells were successfully transduced by each of the retroviral vectors on the basis of coexpression of eGFP from an IRES. (B) Cell cycle analysis of NIH3T3 cells transduced with a retroviral vector (Migr) expressing hGATA-1 or hGATA-1Rb^−^. Transduced cells were synchronized during a 72-h culture in the presence of 1% FCS (serum starvation) and subsequently serum stimulated for 48 h in the presence of 10% FCS. The distribution of cells in G0/G1, S, and G2/M phases was evaluated by measuring their DNA content by cytofluorometry following Hoechst 33342 (Molecular Probes/Invitrogen) staining. The percentage of cells in the different phases of the cell cycle was evaluated as described in Materials and Methods. (C) NIH-3T3 cells transduced with either an “empty” retroviral vector (Migr) or one that encodes hGATA-1 or hGATA-1Rb^−^ were transiently transfected with increasing amounts (1- to 5-fold) of a hFOG-1 expression plasmid and the proliferation of the cells monitored for 5 d by Uptiblue reagent (Day 0 corresponds to the day of the FOG-1 plasmid transfection). We verified that >90% cells were successfully transduced by each of the retroviral vectors on the basis of coexpression of eGFP from an IRES. To increase legibility, curves are numbered 1 to 10 as well as represented with different colors. (D) NIH-3T3 cells, transduced with either an “empty” retroviral vector (Migr) or a retroviral vector that expresses hGATA-1 or hGATA-1^V205G^, were transiently transfected with the same amount of hFOG-1 or hFOG-1^S706R^. We verified that >90% cells were successfully transduced by each of the retroviral vectors on the basis of coexpression of eGFP from an IRES. Cell proliferation was monitored each day for 5 d by Uptiblue (Day 0 corresponds to the day of the FOG-1 plasmid transfection). Two independent experiments gave a similar result (*n* = 6 for each experiment). To increase legibility, curves are numbered 1 to 7 as well as represented with different colors.

As GATA-1 overexpression is known to inhibit cell growth [4],[5], we then set out to study the putative role of the GATA-1/pRb/E2F complex on cell proliferation. Because erythroid cells naturally express both GATA-1 and FOG-1, thus making it difficult to dissect the respective contribution of each of the factors and to decouple their effects on proliferation per se versus terminal erythroid cell differentiation, we first chose to establish a reconstituted cellular model after forced expression of wt and mutant GATA-1 proteins together with their corresponding FOG-1 interacting partners in the nonerythroid NIH3T3 cell line. We monitored, every day for 4 d, the growth of NIH-3T3 cells transduced with retroviral vectors that express either hGATA-1 or hGATA-1Rb^−^. Because hGATA-1 and hGATA-1Rb^−^ proteins were expressed at similar levels in transduced cells (Figure 1B) with undistinguishable transcriptional activity (Figure S3), this assay becomes relevant. Whereas hGATA-1 expression impaired NIH-3T3 cell proliferation, as previously reported [5], we found that hGATA-1Rb^−^ had no effect on it (Figures 3A and S4). Because pRb/E2F has been shown to be necessary to the G1/S transition [25],[26], we investigated whether the cellular distribution of phases of the cell cycle would be altered by the GATA-1/pRb/E2F association. NIH3T3 cells were transduced with retroviral vectors constitutively expressing either hGATA-1 or hGATA-1Rb^−^. Transduced NIH3T3 (eGFP-positive) cells were then starved in 1% serum (cell synchronization) for 72 h, and cell cycle progression was studied after stimulation in 10% serum at Day 2. As previously described [5], expression of GATA-1 blocked the cell cycle at the G1/S transition, whereas hGATA-1Rb^−^ expression did not (Figure 3B).

To clarify the role of pRb in the observed GATA-1–dependent impairment of cell growth, we knocked down pRb expression in the transduced NIH-3T3 cells by transient transfection of a small interfering RNA (siRNA) directed against pRb. Consistent with our expectation, GATA-1–mediated inhibition of cell growth was abrogated for 2 d before cells ultimately stopped proliferating as the inhibitory effect of the siRNA vanished (Figure S5A). pRb knock-down by siRNA was concurrently assessed by western blot analysis, which indicated that complete inhibition of pRb expression occurred only during the first 2 d after siRNA transfection (Figure S5B) without interference with the expression of p107 (Figure S5C). When the same experiment was performed with the human osteogenic sarcoma SAOS-2 cell line, which does not express endogenous GATA-1 or pRb, neither retrovirally expressed hGATA-1 nor hGATA-1Rb^−^ had an effect on cell proliferation (Figure S5D). These results together establish that direct interaction of pRb to GATA-1 through its LXCXE motif is central to the antiproliferative effect of GATA-1.

We then focused on the putative role of FOG-1 in this process. NIH-3T3 cells constitutively expressing hGATA-1 after retroviral transduction were transiently transfected with increasing amounts of a hFOG-1 expression plasmid and cell proliferation monitored for 5 d. hFOG-1 relieved the growth inhibitory effect of hGATA-1 for NIH-3T3 cells in a dose-dependent manner (Figure 3C). When the same experiment was performed with the non–FOG-1–interacting mutant hGATA-1^V205G^ instead of hGATA-1, hFOG-1 was unable to counteract the inhibition of NIH-3T3 cell proliferation mediated by hGATA-1^V205G^ at the displacing dose of FOG-1 established for hGATA-1 (Figure 3D). When hFOG-1^S706R^ was combined with hGATA-1^V205G^ in a similar experiment, rescue of cell proliferation was observed albeit only partially (Figure 3D), consistent with the lower affinity of hFOG-1^S706R^ for hGATA-1^V205G^ compared to that of hFOG-1 for hGATA-1 (Figure 2B and [30]).

**Figure 4:**
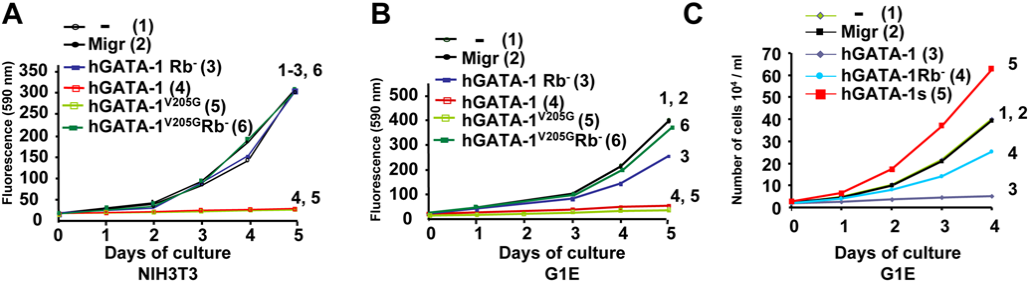
GATA-1, FOG-1, and pRb/E2F-2 interplay regulates proliferation. (A) Cellular proliferation of eGFP-positive NIH 3T3 cells transduced with an “empty” retroviral vector (Migr) or retroviral vectors encoding hGATA-1, hGATA-1Rb^−^, hGATA-1V205G, or hGATA-1V205GRb^−^. We verified that >90% cells were successfully transduced by each of the retroviral vectors on the basis of coexpression of eGFP from an IRES. Cell proliferation was monitored each day for 5 d by Uptiblue. To increase legibility, curves are numbered 1 to 6 as well as represented with different colors. (B) Same as in (A), but using the G1E cell line. Because of the lower retroviral transduction efficiency of G1E cells (∼30%), we first sorted eGFP-positive cells for each of the retroviral vectors used on the basis of coexpression of eGFP from an IRES and then verified that the sorted population was >95% eGFP positive. To increase legibility, curves are numbered 1 to 6 as well as represented with different colors. (C) Same as in (B), but G1E cells were transduced with a retroviral vector (Migr) that expresses GATA-1s , which comprises a deletion of the first 83 amino acids.

To establish the relevance of these finding for erythroid cells, we made use of the erythroid cell line G1E, which is blocked at the proerythroblast stage and derives from mouse embryonic stem (ES) cells with complete biallelic inactivation of the endogenous GATA-1 genes (*GATA-1* ^−*/*−^) [31]. G1E and NIH3T3 cells were transduced with retroviral vector constitutively expressing either hGATA1, hGATA-1Rb^−^, hGATA-1^V205G^, or hGATA-1^V205G^Rb^−^. The hGATA-1^V205G^ mutant and the double hGATA-1^V205G^Rb^−^ mutant were included in this study in an effort to decouple effects on cell proliferation per se versus terminal erythroid cell differentiation, which is known to be dependant upon GATA-1/FOG-1 interaction and would here confuse data interpretation since G1E cells naturally express FOG-1. In agreement with the conclusion we reached in reconstituted NIH3T3 cells, the various GATA-1 forms behaved similarly in G1E and NIH3T3 cells (i.e., inhibition of cell proliferation with hGATA1 and hGATA-1^V205G^, as opposed to maintenance of cell proliferation with hGATA-1Rb^−^ and hGATA-1^V205G^Rb^−^) (Figure 4A and 4B). However, the rate of cell proliferation was lower in the G1E cell population in the presence of the hGATA-1Rb^−^ mutant as compared to G1E cells expressing the double hGATA-1^V205G^Rb^−^ mutant and NIH3T3 cells expressing either of these single or double mutants, in agreement with findings discussed in the subsequent section that the hGATA-1Rb^−^ mutant induces partial terminal erythroid differentiation of the G1E cell population through GATA-1/FOG-1 complex formation. Taken together, these findings establish a novel function for FOG-1 and point to the role of the GATA-1/FOG-1 association as a potential molecular rheostat to regulate the inhibition of cell proliferation induced by GATA-1. However, G1E cells transduced with a retroviral vector that expresses GATA-1s hyperproliferate (Figure 4C). This suggests that hGATA-1Rb^−^, and hGATA-1s are not functionally equivalent.

### Forced Expression of hGATA-1Rb^−^ Fails to Induce Proper Terminal Erythroid Differentiation of GATA-1^−/−^ Cells

**Figure 5:**
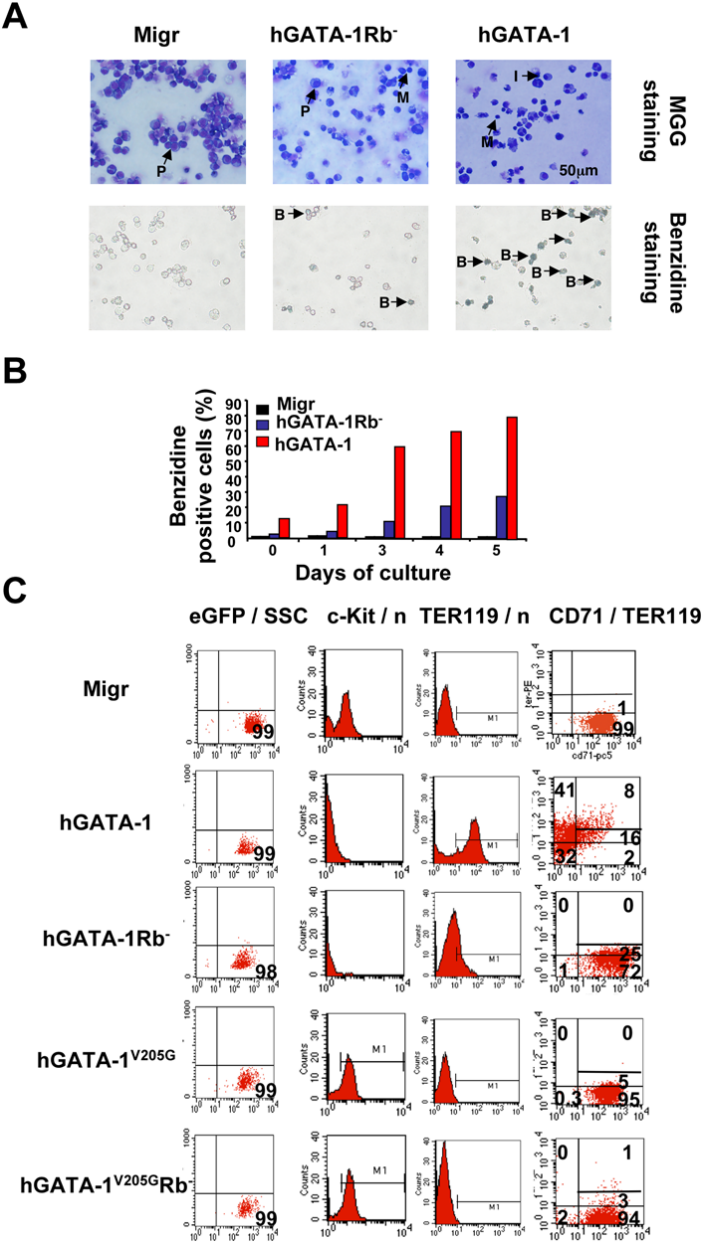
GATA-1, FOG-1, and pRb/E2F-2 interplay regulates the differentiation of G1E cells. (A) GATA-1–deficient G1E cells were transduced with either an “empty” retroviral vector (Migr) or one that encodes hGATA-1 or hGATA-1Rb^−^. Transduced G1E cells were first sorted for eGFP expression. We verified that the sorted population was >95% eGFP positive. Cell differentiation was examined on Day 4 posttransduction by May-Grünwald-Giemsa (MGG) (top) and benzidine (bottom) staining. Proerythroblast, intermediaries, mature erythroblast, and benzidine-positive cells are indicated as P, I, M, and B, respectively. (B) Graphic representation of benzidine-positive G1E cells after retroviral transduction (eGFP-sorted population). The percentage of benzidine-positive cells was monitored for 5 d. (C) Erythroid differentiation of G1E cells transduced with an empty retroviral vector (Migr) or one that expresses hGATA-1, hGATA-1Rb^−^, hGATA-1^V205G^, or hGATA-1^V205G^Rb^−^ was assessed, after 6 d in culture, by flow cytometry analysis for c-Kit, TER119, and CD71 expression. Transduced G1E cells were first sorted for eGFP expression. Panels on the left column confirmed that sorted cells were >98% positive for eGFP expression. The *x-* and *y-*axes are indicated at the top of the panels (SSC = side scatter; *n* = cell counts). For panels in the left column, TER119^high^ and TER119^low^ are delineated.

Since pRb has been implicated in the differentiation processes of several cell types [16],[26], in addition to its role in cell cycle control, we then investigated whether the interaction between pRb and GATA-1 is involved in the differentiation of erythroid cells. To this end, we returned to the erythroid cell line G1E. Retrovirus-mediated expression of hGATA-1 in G1E cells induced terminal erythroid differentiation of cells positive for coexpression of vector-encoded eGFP, as evidenced by May-Grünwald-Giemsa and benzidine staining for identification and scoring of the various red blood cell precursors (Figure 5A and 5B). In contrast, forced expression of hGATA-1Rb^−^ dramatically altered the distribution of erythroid precursors towards the more immature elements, although the initiation of erythroid differentiation was not impaired (Figure 5A and 5B). The same results were obtained with another erythroid cell line, referred to as GAK14 (M. Yamamoto, unpublished data), which was independently developed from mouse GATA-1.05 ES cells (Figure S6A). To determine more precisely the cell composition of G1E-transduced (eGFP positive) cells, erythropoietic maturation was assessed 6 d after transduction by cytofluorometry for c-Kit, CD71, and TER119 expression. Although the complete loss of c-Kit expression in both hGATA-1– and hGATA-1Rb^−^–transduced G1E cells indicated that erythroid differentiation was initiated normally in either case, quantification of CD71^−^TER119^hi^ (41% vs. 0%) and CD71^hi^TER119^−^ (2% vs. 72%), respectively, showed that terminal erythroid differentiation was impaired in hGATA-1Rb^−^ G1E cells (Figure 5C). GATA-1Rb^−^ G1E cells were able to progress to an intermediate (CD71^hi^TER119^lo^) stage like hGATA-1 G1E cells (25% vs. 16%, respectively, Figure 5C), but hGATA-1 G1E cells differentiated further. A similar conclusion was reached by morphological identification and scoring of the various red blood cell precursors of the retrovirally transduced GAK14 cell line (Figure S6B).

To study the potential effects of the interplay between GATA-1, pRb/E2F, and FOG-1 upon the differentiation of erythroid cells, we analyzed by flow cytometry the phenotypes of G1E cells expressing hGATA-1, hGATA-1^V205G^, or hGATA-1^V205G^Rb^−^ after retroviral vector transduction (Figure 5C). Whereas hGATA-1–expressing cells became c-Kit^−^, CD71^+^, and TER119^+^, both hGATA-1^V205G^ and hGATA-1^V205G^Rb^−^ expressing cells failed to express TER119 and continued to express c-Kit. These data indicate that it is the GATA-1/FOG-1 association, but not the GATA-1/pRb/E2F complex, which is required for the down-expression of c-Kit during the early stage of proerythroblast differentiation.

### Direct Interaction of GATA-1 with pRb Is Necessary for Terminal Erythropoiesis In Vivo

**Figure 6:**
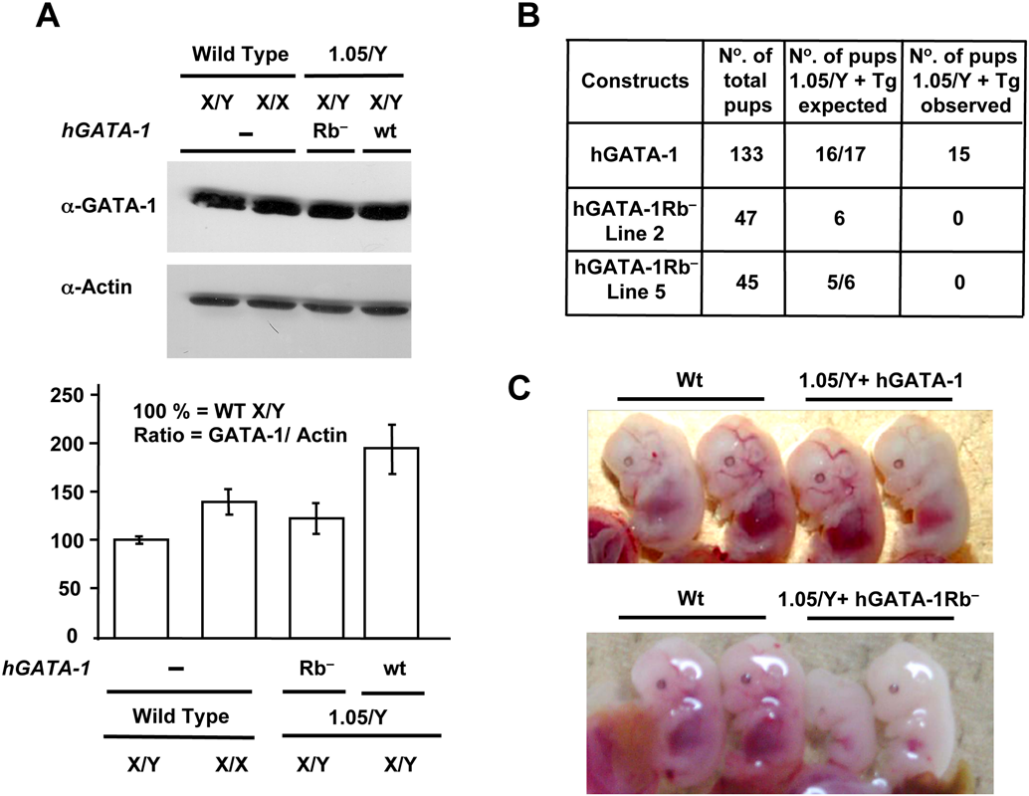
GATA-1/pRb interaction is necessary for terminal erythropoiesis in vivo. (A) Comparative levels of GATA-1 proteins in fetal livers of E12.5 wt or transgene-expressing mouse embryos. Top, fetal livers from one wt male (X/Y) and one wt female (X/X) E12.5 embryos as well as from two males *GATA-1.05*/*Y* (referred to as “1.05/Y” in the figure) transgenic for either hGATA-1 (referred to as “wt” in the figure) or hGATA-1Rb^−^ (referred to as “Rb^−^” in the figure). Nuclear extracts were subjected to western blot analysis using an α-GATA-1 Ab and reprobed with an anti–β-actin (α-Actin) Ab (AC-15 clone; Sigma). Bottom, GATA-1 protein levels were quantified by chemiluminescence (Intelligent Dark box, LAS-3000, Fujifilm) and normalized for β ˜-actin protein levels (*n* = 3). Results are expressed as means±standard deviation (SD) of three independent experiments. (B) Crossbreeding of *XGATA-1.05/X* female mice with transgenic male mice expressing hGATA-1 or the mutant hGATA-1Rb^−^ proteins results in four possible genotypes with or without the transgene. Since the *GATA-1* gene is located on the X chromosome, hemizygous *XGATA-1.05/Y* mice die in utero. The expected (if the transgene was resulting in complete rescue) and the observed pup number of *XGATA-1.05/Y* mice harboring the transgenes are indicated. (C) Top, fetal livers from E13.5 *GATA-1.05/Y+hGATA-1wt* control embryos (referred to as “1.05/Y+hGATA-1wt” in the figure) show an indistinguishable appearance from their wt littermates (Wt), and viable pups were born with the expected Mendelian distribution. Bottom, in contrast, *GATA-1.05/Y+hGATA-1Rb* ^−^ embryos in lane 5 (referred to as “1.05/Y+GATA-1Rb^−^” in the figure) are pale comparatively to their wt littermates (Wt) and die in utero.

To assess the putative physiological effects of the protein–protein interaction between GATA-1 and pRb in a whole animal, we performed a transgenic complementation rescue assay [32]. Because the *GATA-1* gene is located on the X chromosome, which is randomly inactivated in every cell, neither homozygous *GATA-1* ^−^ */GATA-1* ^−^ females nor hemizygous *GATA-1* ^−^ */Y* males are viable. We thus made use of mice that carry a mutant *GATA-1* allele, referred to as *GATA-1.05*, which expresses only 5% of GATA-1 wt levels. As previously shown, male *GATA-1.05*/*Y* mice die in utero from impaired hematopoiesis, whereas heterozygous female mice (*GATA-1.05*/*X*) spontaneously recover shortly after birth from embryonic/fetal and neonatal anemia [32]. In preparation for crossing experiments with these female mice, we generated transgenic (Tg) lines of mice that express either hGATA-1Rb^−^ or its wt counterpart hGATA-1 under the control of transcriptional regulatory sequences referred to as GATA-1 hematopoietic regulatory domain (HRD). HRD-driven transcription is known to recapitulate the endogenous *GATA-1* gene expression pattern in both primitive and definitive erythroid lineages of Tg mice [1]. We focused on two of the hGATA-1Rb^−^–expressing lines (Tg Lines 2 and 5), because expression of the *hGATA-1Rb* ^−^ transgene was at a level equivalent to that of the endogenous mouse *GATA-1* gene (Figure 6A). Mouse Tg line 8 was also analyzed as an example of supraphysiologic expression of the *hGATA-1Rb* ^−^ transgene (level 280% that of the endogenous mouse *GATA-1* gene). Males of these Tg mice were then mated with heterozygous *GATA-1.05/X* females, and their male progeny, referred to as *1.05/Y+hGATA-1Rb* ^−^ and *1.05/Y+hGATA-1*, studied. The expected and the observed pup numbers of *XGATA-1.05/Y* mice harboring the transgene are indicated in Figure 6B. The first observation we made is that the control *hGATA-1* transgene could fully rescue *GATA-1.05/Y* male mice from lethality. Although some of the *1.05/Y+hGATA-1* male embryos showed a slightly anemic appearance at E13.5, most were indistinguishable from wt littermate embryos (Figure 6C). Importantly, their erythropoiesis caught up during late gestation, and viable pups were born with the expected Mendelian distribution (15 rescued out of 133 pups). In contrast, *1.05/Y+hGATA-1Rb* ^−^ males from either Tg Line 5 (0 rescued out of 45 pups) or Tg Line 2 (0 rescued out of 47 pups) suffered from profound anemia and small size at E12.5–E13.5 leading to embryonic lethality for all animals around E15.5 of development (Figure 6C). In contrast and as expected from previous studies of other GATA mutants, mice from Tg Line 8, which expressed supraphysiologic levels of *hGATA-1Rb* ^−^, were indistinguishable from wt littermate embryos. This observation underscores the importance of studying the phenotypic effects of mutant GATA transgenes within a physiologic range of expression for this type of transgenic mouse approach (see Discussion).

**Figure 7:**
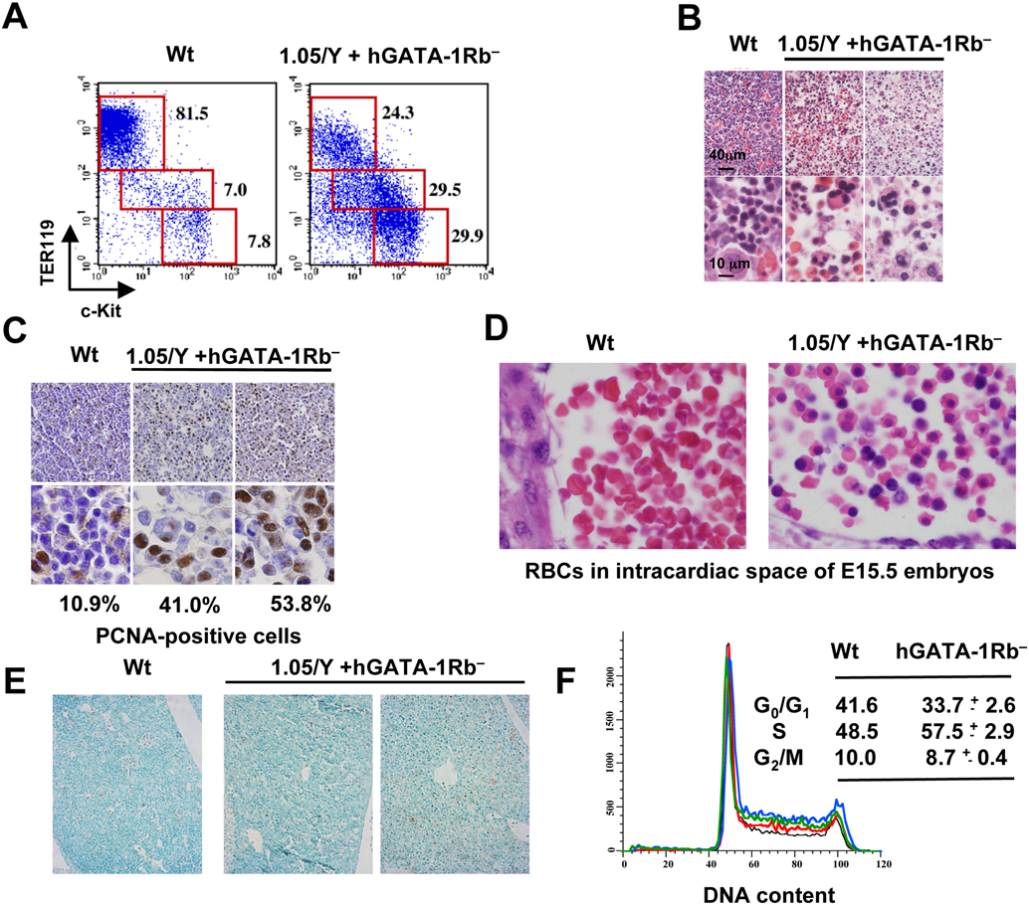
Analysis of fetal hematopoiesis in 1.05/Y+hGATA-1Rb^−^ mice. (A) Analysis by flow cytometry of liver cells from E13.5 Wt or 1.05/Y+hGATA-1Rb^−^ embryos. Cells were labeled with c-Kit and TER119 Abs, showing a decrease in the proportion of TER119^+^/c-Kit^−^ mature erythroblasts with compensatory increase in proerythroblasts and earlier erythroid precursors. (B) Liver sections from E15.5 wt or 1.05/Y+hGATA-1Rb^−^ embryos stained with hematoxylin and eosin show a decrease in the number of erythroid cells (top) with evidence of abnormal erythroblasts having lobulated nuclei at higher magnification (bottom). (C) Immunohistochemical analysis for Proliferating Cell Nuclear Antigen (PCNA) in liver sections from E12.5 wt or 1.05/Y+hGATA-1Rb^−^ embryos. High magnification panels (bottom) show that the proportion of PCNA-positive cells among erythroblasts is higher in 1.05/Y+hGATA-1Rb^−^ embryos than in wt embryos. (D) Hematoxylin and eosin analysis of red blood cells in the intracardiac space of E15.5 embryos. (E) Immunohistochemical analysis for Terminal dUTP Nick-End Labeling (TUNEL) assay to detect apoptotic cells in liver sections from E12.5 wt or 1.05/Y+hGATA-1Rb^−^ embryos. (F) Cell cycle distribution in hGATA-1Rb^−^ mice. DNA content analysis of CD71-positive cells from livers of e12.5 wt (black line) and individual 1.05/Y+GATA-1Rb^−^ embryos (red, green, and blue lines). Percentages of each subpopulation of cells in G_0_/G_1_, S, and G_2_/M phases are indicated.

Flow cytometry analysis for c-Kit and TER119 expression of liver cells from *1.05/Y+hGATA-1Rb* ^−^ male E13.5 embryos generated from either Lines 2 or 5 showed a decrease in the proportion of mature erythroid cells and late erythroid precursors with concurrent increase in that of early precursors, comparatively to wt littermate embryos (24% vs. 81%^−^ for TER119^high^ c-Kit^low^ and 30% vs. 8% for TER119^−^ c-Kit^high^, respectively), indicating a severe delay in erythroid maturation (Figure 7A). No significant delay was observed in *1.05/Y+hGATA-1* embryos at E13.5 (unpublished data). The liver of E15.5 embryos also contained abnormal erythroid precursors with lobulated nuclei (Figure 7B). Staining of fetal liver sections for the proliferating cell nuclear antigen (PCNA) revealed an increased number of mitotic cells (Figure 7C). Red blood cells harvested from the intracardiac space of E15.5 GATA-*1Rb* ^−^ embryos showed defective erythroid terminal differentiation illustrated by a significant proportion of nucleated cells with defects in nuclear condensation (Figure 7D) and a mild increase in the number of apoptotic cells (Figure 7E). Furthermore, analysis of CD71-positive cells showed a mild accumulation of cells in the S phase of the cell cycle (Figure 7F). These features share striking similarities to those observed in the fetal liver cells of pRb^−/−^ mice [33] and with our data obtained with transduced G1E and GAK14 cell lines (Figures 5 and S6, respectively).

## Discussion

We have shown here that (1) direct protein–protein interaction between GATA-1 and pRb is required for normal terminal erythroid differentiation in vitro and in vivo, (2) in late-stage erythroid cells from mouse fetal liver, GATA-1 associates, through a LXCXE motif, with hypophosphorylated pRb engaged with E2F-2 to form a GATA-1/pRb/E2F-2 tri-complex, (3) GATA-1 and pRb do not form a bicomplex in the absence of E2F-2, (4) the GATA-1/pRb/E2F-2 tricomplex inhibits the proliferation of erythroid precursors (5) the association of GATA-1 to pRb/E2F alters G1-to-S phase progression and (6) FOG-1 is capable of displacing pRb/E2F-2 in vitro from the GATA-1/pRb/E2F-2 tricomplex. The physiological relevance of this latter observation needs to be further investigated.

A mechanism by which GATA-1/pRb/E2F-2 tricomplex induces cell cycle arrest during erythroid differentiation is likely to be, at least in part, by sequestering E2F within a complex in which pRb is in its hypophosphorylated form. FOG-1 is able to displace GATA-1 away from the complex, and this may then allow E2F-2 to be liberated upon subsequent phosphorylation of pRb. In parallel, the GATA-1/FOG-1 complex exerts its transcriptional effects as part of the program of erythroid differentiation, as exemplified by the extinction of c-Kit expression. Fine-tuning by exogenous inducers is likely to operate, as pRb and GATA-1 are regulated by phosphorylation [34]. This study does not yet address the possibility that the GATA-1/pRb/E2F-2 tricomplex possesses specific transcriptional activity in addition to the mere sequestration of E2F-2. For instance, GATA-1/pRb/E2F-2 may activate or inhibit E2F target genes transcription by (1) displacing other pRb-associated LXCXE proteins, (2) increasing gene transactivation through the GATA-1 transactivator domain, (3) acting as a protein platform for other associated GATA-1 proteins (e.g., acetyltransferase P300/CBP, PU.1, EKLF). Preliminary chromatin immunoprecipitation (ChIP) assays, using a GATA-1–specific Ab for ChIP, indicate that known E2F targets genes such as Cdc6 [35] can be detected (unpublished data). Transcriptional studies and cDNA microarrays analysis would sort through these hypotheses, as previously conducted for other Rb-associated proteins that include Id2 [36].

For the past several years, conflicting reports have sparked an active debate as to whether the fatal anemia observed in *Rb* ^−*/*−^ mice was of extrinsic (cell-nonautonomous) [16],[17] and/or intrinsic (cell-autonomous) [18],[19] origin. During the revision of this manuscript, two studies have added to our understanding of the role of pRb during erythroid differentiation. The Walkley and Orkin laboratories have gathered convincing evidence for a cell-type–intrinsic and cell-autonomous role of pRb in erythropoiesis, using conditional *Rb* inactivation restricted to the erythroid lineage [21]. Another study from the Macleod laboratory has shed light on the interplay between pRb and E2F-2 during erythropoiesis by showing that the concurrent loss of E2F-2 and pRb is surprisingly capable of rescuing terminal erythroid maturation to Rb null red cells [20]. With the double mutants, erythroid precursors resume normal cycle cell arrest in S phase and concurrent terminal erythroid maturation, although the compensatory mechanisms remain unknown [20]. Our study brings a molecular basis for the existence of an intrinsic component by establishing a direct physical link between pRb/E2F-2 and the master transcription factor of erythroid differentiation GATA-1.

Our findings also provide an explanation for the lack of full rescue of GATA-1–deficient mice by the related factors GATA-2 and GATA-3 when expressed in lieu of GATA-1, since GATA-2 and GATA-3, which share extensive functional similarities with GATA-1 [37], do not contain an LXCXE motif. The variable degree of severity of the observed phenotypes between various studies is likely to reflect the levels of expression obtained for the GATA-2 and GATA-3 factors according to the transgenic approach used, ranging from embryonic lethality by lack of erythropoiesis to viability with anemia at the adult stage [32],[37]. A similar observation has been reported with other GATA-1 mutants overexpressed in the same transgenic mouse model [38], indicating the importance of comparing GATA factors within the same range of transgene expression levels in these studies [39]. So is our observation with the supraphysiologic expression level of the *hGATA-1Rb* ^−^ Tg Line 8, and it is likely that pRb-independent pathways are also affected in the case of GATA overexpression. For instance, supraphysiologic GATA-1 expression levels may disturb the optimal GATA-1 to Gfi-1B cellular ratio, thus deregulating the antiapoptotic Bcl-x(L) factor [40], or alter microRNA (miRNA) levels during erythroid maturation [41].

In humans, a severe anemia has been recently described in a family of patients with an inherited splicing mutation of the *GATA-1* gene that results in exon skipping and expression of an N-terminally deleted GATA-1 protein (GATA-1s) [23]. GATA-1s is translated from a downstream ATG initiation codon that encodes Met^84^ in the full-length GATA-1. Here, we show that this mutation disrupts the association of GATA-1s to pRb because Met^84^ is located within the LNCM^84^E motif, thus deleting it from the GATA-1s form. However, our data also suggest that GATA-1s and hGATA-1pRb^−^ are not functionally equivalent and, thus, that it is likely that the N-terminal moiety of GATA-1 has an additional Rb-independent role. hGATA-1s may thus result in a phenotype less severe than hGATA-1pRb^−^ possibly because a double loss of function may elicit a syndrome of lesser severity than a single loss, as observed with the double-null mice E2F-2^−/−^ pRb^−/−^ [20] or Id2^−/−^ pRb^−/−^ [16]. A caveat to the interpretation of these experiments is that the mutation in the LXCXE motif may concurrently disrupt some other critical yet unknown function of GATA-1. These observations may also help understand the specific pre-leukemic syndrome associated with acquired somatic GATA-1 mutations, which also result in GATA-1s expression, in patients with the Down syndrome (trisomy 21) [24].

The interplay uncovered here between factors that regulate cell cycle and transcription factors key to the differentiation of the red blood cell lineage should be considered for the interpretation of pRb, GATA-1, or FOG-1 mutant mice and corresponding mutations associated with specific human syndromes. These findings may also form the basis for the discovery of similar pathways in other tissues during both ontogenesis and homeostatic cell production throughout life.

## Materials and Methods

### Plasmid Construction and Retroviral Transduction

Human GATA-1Rb^−^ cDNA was generated by PCR and sequenced. This cDNA was then subcloned in the retroviral vector Migr. Human FOG-1 cDNA was subcloned in pRSVcDNA3 (Invitrogen). GATA-1^V205G^, GATA-1^V205G^Rb^−^, and FOG-1^S706R^ mutants were obtained using the QuickChange Site-Directed Mutagenesis Kit (Stratagene) with respective, previously described, plasmids as templates. Construct sequences were confirmed by DNA sequencing. Retroviral production and cell transduction were performed as previously described [34]. Two days after transduction, GFP-positive cells were sorted by flow cytometry (Epics Altra; Beckman Coulter).

### Cell Culture and Transfection

NIH-3T3 were maintained at low population doubling and density. NIH-3T3 and SAOS-2 cells were transfected using lipofectamine 2000 transfection reagent (Invitrogen). Uptiblue reagent (Interchim) was used for cell proliferation assays with fluorimetric excitation at 560 nm and reading at 590 nm (Typhoon 9400; Amersham Bioscience). G1E cells were grown as previously described [34]. Cytospin samples were stained with May-Grünwald-Giemsa to assess and score the various stages of erythroid differentiation. Hemoglobinization was evidenced by benzidine staining. Erythroid cells from mouse fetal livers were obtained from E12.5 C57BL/C embryos.

### Protein Analysis

For nuclear extract preparation, cells were washed once with PBS and incubated for 10 min at 4°C in buffer A (10 mM HEPES [pH 7.6], 3 mM MgCl_2_, 10 mM KCl, 5% glycerol, 0.5% NP-40) containing phosphotyrosine phosphatase inhibitors (1 mM Na_2_VO_4_), phosphoserine/threonine phosphatase inhibitors (20 mM NaF, 1 mM sodium pyrophosphate, 25 mM β-glycerophosphate), and proteinase inhibitors. After centrifugation, nuclear pellets were resuspended in buffer A containing 300 mM KCl. and incubated for 30 min at 4°C. After centrifugation, nuclear extracts were quantified by BCA staining (Pierce), half-diluted with buffer A and subjected to IP using the appropriate Ab: hGATA-1 C-terminal region epitope C-20 (Santa Cruz Biotechnology), mGATA-1 C-terminal region epitope, M-20 (sc-1234; Santa Cruz Biotechnology), pRb (N° 554136; BD Pharmingen), E2F-2 (sc-633; Santa Cruz Biotechnology), FOG-1 (sc-9361; Santa Cruz Biotechnology), Nph (sc-6013R; Santa Cruz Biotechnology), or with nonimmune corresponding (control) Abs, respectively, normal goat IgG (sc-2028), normal mouse IgG (sc-2025), and normal rabbit IgG (sc-2027) (Santa Cruz Biotechnology). Beads were then washed five times with buffer A and resuspended in 1× Laemmli buffer. Western blot analyses were performed as described [34] with primary Abs against GATA-1 (N1, Cat. N° sc-266; Santa Cruz Biotechnology), FOG-1 (M-20, Cat. N° sc-9361; Santa Cruz Biotechnology), pRb (Cat. N° 554136; BD Pharmingen; and human c-terminal pRb epitope C15, Cat. N° sc-50; Santa Cruz Biotechnology) or phospho-specific pRbs (pRb^Pser780^, pRb^Pser870/811^, and pRb^Pser795^ PhosphoPlus Rb antibody Kit Cat. N° 9300; Cell Signaling Technology).

### Generation of Transgenic Mice

Wt and mutant human GATA-1 cDNAs were cloned 3′ to the mouse GATA-1 HRD [32]. DNA fragments were purified from vector sequences and transgenic mice generated by DNA microinjection into fertilized BDF1 eggs using standard procedures. The *GATA-1.05* allele was monitored by PCR using primers corresponding to the neomycin-resistance gene in the original *GATA-1.05* targeting vector.

### PCNA Staining

Whole embryos were fixed in 4% formaldehyde solution at 4°C for 16 h followed by embedding in paraffin. PCNA staining was performed using a kit from Zymed Laboratories.

### Flow Cytometry

Phycoerythrin-conjugated anti-mouse TER119 (TER119-PE; BD Pharmingen, cat N°553673), APC-conjugated anti-mouse CD117 (c-Kit) (BD Pharmingen, cat N°553356), biotin-conjugated anti-mouse CD71 (BD Pharmingen, cat N°557416) and streptavidin-PE-PC5 secondary antibodies (BD Pharmingen) were used for surface labeling of cells. Flow cytometry was performed using FACSCalibur, and data were analyzed with the Cell Quest Pro software.

### Analysis of Cell Cycle Distribution

Cells were pelleted, fixed in 70% ethanol, and then resuspended at a concentration of 10^6^ cells per milliliter in PBS containing 5 mM EDTA and 5 µg/ml Hoechst 33342 (Molecular Probes–Invitrogen). The cells were then analyzed with a LSRII cytometer (BD Biosciences) equipped with both the DIVA and the Flowjo 8.8.3 software using the Dean-Jett-Fox algorithm.

## Supporting Information

Figure S1 **GATA-1 interaction with endogenous pRb and antibody controls.** (A) Human and murine GATA-1 proteins interact with endogenous murine pRb. NIH-3T3 cells were transduced with the “empty” retroviral vector (Migr) or with retroviral vectors encoding either human (h) or murine (m) GATA-1. We verified that >90% cells were successfully transduced by each of the retroviral vectors on the basis of coexpression of eGFP from an internal ribosome entry site (IRES). IPs of GATA-1 (5 µg, α-GATA-1: M-20, sc-1234; Santa Cruz Biotechnology) or pRb (5 µg, α-pRb, n° 554136; BD Pharmingen) were performed with 500 µg of nuclear extract. IPs using 5 µg of nonimmune IgG isotype of the corresponding species (normal goat IgG sc-2028 for GATA-1 and normal mouse IgG sc-2025 for pRb; Santa Cruz Biotechnology) were used for each transfection as negative controls for the specificity of the precipitation and coprecipitation obtained with each immune antibody used in the IP and co-IP experiments. Nuclear extracts before IP (input 5%) and bound proteins were resolved by western blot analysis using the antibody against GATA-1 (α-GATA-1 M-20, sc-1234; Santa Cruz Biotechnology) or pRb (α-pRb, n° 554136; BD Pharmingen) as indicated. The horseradish peroxydase-conjugated secondary Ab used (Affinipure goat anti-rat IgG light chain specific, 112-035-175; Affinipure goat anti-mouse IgG light chain specific, 115-035-175) was provided from Jackson Immunoresearch and did not recognize reduced denaturated IgG heavy chains that comigrate at 50 kDa near GATA-1 (http://www.jacksonimmuno.com/catalog/CatPages/rblc.asp). These secondary Abs were used in all the blotting assays of this paper. This experiment indicates that both human and murine GATA-1 interact with endogenous murine pRb with the same efficiency. (B) Control of the specificity of the Abs utilized. NIH-3T3 cells were transfected with the “empty” retroviral vector (Migr) or with retroviral vectors encoding for either hGATA-1, hGATA-1Rb^−^, or hGATA-1s. IP of GATA-1 (α-GATA-1 M-20, sc-1234; Santa Cruz Biotechnology) or pRb (α-pRb; BD Pharmingen) were performed on 500 µg of nuclear extract. Nonimmune IgG (mouse for pRb and goat for GATA-1; Santa Cruz Biotechnology) were used as controls for each transfection (lanes 2, 7 12, 17, and 4, 9, 14, 19, respectively). Nuclear extracts before IP (input 5%, lane 1, 6, 11, and 16) and bound proteins were resolved by western blot analysis using the Ab against GATA-1 (α-GATA-1 N-1 sc-266; Santa Cruz Biotechnology) or pRb (phospho-specific pRbs: pRbPser780, pRbPser870/811, and pRbPser795 PhosphoPlus Rb antibody Kit Cat. N° 9300; Cell Signaling Technology) as indicated. We thus conclude that only wt GATA-1 can interact with pRb as shown by the complex coprecipitated by GATA-1 antibody as well as the pRb antibody. This interaction is specific as no complex was found in the IgG control lane. Neither GATA-1Rb^−^ nor GATA-1s can interact with pRb (even with a large amount of protein), indicating that the LNCME motif is required for the interaction.
(2.13 MB TIF)

Figure S2 **GATA-1 forms a complex with pRb and E2F-2 in primary erythroid cells.** (A) Experimental procedures used for (B–D). Fetal livers were dissected from C57BL/6 embryos at embryonic day 12.5 (E12.5) and disaggregated in α-MEM supplemented with phosphotyrosine phosphatase inhibitors (1 mM Na_2_VO_4_; Sigma), phosphoserine/threonine phosphatase inhibitors (20 mM NaF, 1 mM sodium pyrophosphate, 25 mM β-glycerophosphate; Sigma), and proteinase inhibitors (Roche) to a single-cell suspension by serial passage through a 23-ga needle, followed by a 27-ga needle. Viability of cells was assessed by Trypan blue staining (<0.5% of positive cells), and erythroid differentiation stages were checked by cell surface labeling using the following Abs: phycoerythrin-conjugated anti-mouse TER119 (TER119-PE; BD Pharmingen, cat N°553673), APC-conjugated anti-mouse CD117 (c-Kit; BD Pharmingen, cat N°553356), biotin-conjugated anti-mouse CD71 (BD Pharmingen, cat N°557416), and streptavidin-PEPC5 secondary antibodies (BD Pharmingen). Flow cytometry was performed on an aliquot of 10^5^ cells using FACScan, and data were analyzed with the DIVA software. Eighty-five percent to 90% of fetal liver cells were CD71^+^ TER119^+^ c-Kit^−^, showing a majority of cells at the late stage of erythroid differentiation. Nuclear extract were prepared as described in Materials and Methods except that the glycerol concentration was 10% instead of 5% to allow for an improved recovery of nuclear pellets. IPs were then performed on equal amounts of nuclear extract (500 µg per point) using specific Abs or nonimmune-related (control) Abs. Immunoprecipitated proteins (bound, lanes 2 and 4) were analyzed by western blot (WB) as well as a 5% fraction of nuclear extract before (input, lane 1) and after (supernatant, lane 3 and 5) each IP. Nucleophosmin (Nph), which does not bind to GATA-1 or pRb complexes, was used as a negative control of the specificity of coprecipitation (lane 6). (B) Efficiency and specificity of immunoprecipitating Abs. IPs were performed using nuclear lysates from E12.5 fetal livers using specific Abs against GATA-1 (sc-1233; Santa Cruz Biotechnology), pRb (554136; BD Pharmingen), E2F-2 (sc-633; Santa Cruz Biotechnology), FOG-1 (sc-9361; Santa Cruz Biotechnology), and Nph (sc-6013R; Santa Cruz Biotechnology), or with nonimmune corresponding (control) Abs, respectively, normal goat IgG (sc-2028), normal mouse IgG (sc-2025), and normal rabbit IgG (sc-2027) (Santa Cruz Biotechnology). Proteins were analyzed by western blot using the same Abs (except for GATA-1, where the sc-266 from Santa Cruz Biotechnology was used). As expected, all the proteins were totally immunodepleted from the nuclear extracts, and no signal was detected in the supernatants after each IP (lane 4 versus 5). This depletion thus demonstrates specific antigen–Ab interactions, since no protein is immunoprecipitated by the related control Ab (lane 2) or by a nonspecific Ab (Nph, lane 6). (C) Identification of GATA-1 complexes in fetal liver erythroid cells and relevant Ab controls in co-IP assays. Co-IPs were performed using nuclear lysates from E12.5 fetal livers, as described in (A). The first step of IP was performed with either a specific Ab directed against GATA-1 (sc-1233; Santa Cruz Biotechnology), a nonimmune corresponding Ab (control Ab: normal goat IgG sc-2028), or nonspecific anti-Nph Ab. Coprecipitated proteins were then analyzed by western blot using the same Abs as in (B), referred at the left-hand side of the figure. None of the proteins studied is unspecifically coprecipitated with the nonimmune idiotype (lane 2) or a nonspecific antibody (lane 6) even in the presence of a large amount of proteins. As expected, FOG-1, a known partner of GATA-1, is coprecipitated and serves as a positive control (lane 4). Endogenous pRb and E2F2 are also copurified with GATA-1 in a specific manner (lanes 4b and 4c). Note that a small portion of each fraction remains unassociated with GATA-1, corresponding to free proteins or to distinct complexes that exclude GATA-1 (lane 5). (D) Identification of E2F2 complexes in fetal liver erythroid cells and relevant Ab controls in co-IP assays. Same as in (C), except that the first step of IP was performed with either a specific Ab directed against E2F-2 (sc-633; Santa Cruz Biotechnology), a nonimmune corresponding Ab (control Ab: normal rabbit IgG sc-2027), or nonspecific anti-Nph Ab. As obtained in (C), pRb, the E2F-2 known partner, is copurified by E2F-2 immunodepletion (lane 4b), and we show that GATA-1 is also coprecipitated (lane 4a). These observations allow us to propose the existence of a ternary complex containing pRb, GATA-1, and E2F-2. This complex excludes FOG-1: no FOG-1 protein is observed in lane 4d, and the totality of FOG-1 is recovered in the supernatant (lane 5d versus lane 3d). As a faint amount of GATA-1 and pRb remain in the supernatant (lane 5a), this experiment cannot in and of itself exclude the possibility that GATA-1 and pRb can form a binary complex without E2F-2. The convincing evidence that GATA-1 and pRb do not form a bicomplex in the absence of E2F-2 is provided in Figure 1D. (E) Experimental procedure of Figure 1D.
(3.13 MB TIF)

Figure S3 **hGATA-1 and hGATA-1Rb** ^−^ **proteins have undistinguishable transcriptional activity.** To assess the respective transcriptional activities of hGATA-1, hGATA-1Rb^−^, and hGATA-1s, we performed transient transfection assays in NIH-3T3 cells using a luciferase reporter gene driven by several minimal erythroid-specific promoters that included (1) the erythroid porphobilinogene deaminase promoter (nucleotides −714 to +78 PBGD) [42], (2) the glycophorin-B promoter (−95GpB) [43], (3) the erythropoietin receptor promoter (−58, +43 Epo-R) [44], (4) the 3XGATA-TK synthetic promoter [45], and (5) the GATA-1 proximal promoter (IE) together with its upstream enhancer element (IE3.9int) [46]. These different plasmids were cotransfected with the “empty” Migr plasmid or with Migr-derived plasmids expressing hGATA-1, hGATA-1Rb^−^, or hGATA-1s. Firefly luciferase activity was measured according to the manufacturer's instructions (Dual Luciferase Reporter Assay System; Promega), and individual transfections were normalized by quantification of Renilla luciferase activity (pRL-TK; Promega). The total amount of DNA was kept constant at 700 ng in each transfection (500 ng of expression plasmid, 100 ng of reporter plasmid, and 100 ng of pRL-TK plasmid per well of a 24-well plate). Luciferase activity was determined 24 h after transfection. Data are expressed as relative luciferase activity (RLu). Results are the means±standard error of the mean (SEM) of three independent experiments. No difference in the transcriptional activity of hGATA-1 and hGATA-1Rb^−^ was detected with these promoters. With respect to hGATA-1s, the Epo-R minimal erythroid (pEpoR) and the GATA-1 (IE3.9int) promoters were activated at a lower level (approximately 50%), whereas the erythroid porphobilinogene deaminase promoter was not activated, and both the glycophorin-B (−95GpB) and the synthetic 3XGATA-TK promoters were equally transactivated.
(1.07 MB TIF)

Figure S4 **Comparison of the two different methods used to monitor cell proliferation.** Retroviral vectors encoding hGATA-1 or the hGATA-1 mutant that cannot interact with pRb (hGATA-1Rb^−^) were used to transduce NIH-3T3 cells (100% eGFP-positive cells). Proliferation of the transduced NIH-3T3 cells was monitored each day for 5 d. For cell growth analysis, identical numbers of NIH-3T3 cells transduced with retroviral vectors expressing hGATA-1 or hGATA-1Rb^−^ were plated on Day 1. (A) At each indicated day, the number of viable cells was determined by trypan blue dye exclusion (*n* = 3). Mock indicates the “empty” Migr retroviral transduction. (B) Representative growth curve obtained by using the fluorimetric metabolic growth indicator Uptiblue reagent (*n* = 3). (C) Comparison of the analysis obtained with the two methods used in (A) and (B): each data point obtained in (A) was converted into a percentage of cell numbers comparatively to the initial number of cells at Day 0 (top). Each data point obtained in (B) was converted into a percentage of the 590-nm fluorescence in arbitrary units comparatively to that measured at Day 0 (bottom). The resulting curves and the *R* ^2^ values were determined by the Excel software (Microsoft). The observation that the deduced *R* ^2^ curves are of identical value indicates that the two methodologies are comparable. The proliferation of NIH-3T3 cells can be thus monitored by either trypan blue dye exclusion or Uptiblue reagent.
(1.78 MB TIF)

Figure S5 **GATA-1/pRb interaction is required for GATA-1–mediated arrest of cell proliferation.** (A) Same as in Figure 3A, except that the hGATA-1Rb^−^ retrovirus was not used here and that proliferation was monitored in the presence of a siRNA directed against endogenous pRb. The siRNA was transfected at Day 0 of the experiment (*n* = 4). (B) Retroviral vectors expressing hGATA-1 or hGATA-1Rb^−^ were used to transduce NIH-3T3 cells in the absence (−) or in the presence (+) of an siRNA directed against endogenous pRb (sc-29469; Santa Cruz Biotechnology). pRb knock-down analysis was performed each day for three consecutive days. Total proteins were subjected to western blot analysis using an anti-pRb antibody (BD Pharmingen). (C) Membranes were then stripped and reprobed with an anti-p107 antibody (sc 318; Santa Cruz Biotechnology). These data indicate that pRb protein levels were very low shortly after transfection of the siRNA directed against pRb and started to increase 3 d thereafter. The level of pRb expression at Day 3 correlates with the decrease in cell proliferation observed the same day with NIH-3T3 cells transduced with a retroviral vector expressing hGATA-1 and transfected with the pRb siRNA [see (A)]. (D) The same retroviral vectors as in (A) were used to transduce the human cell line SAOS-2, which expresses neither GATA-1 nor pRb endogenously. Cell proliferation was monitored each day for 4 d with the Uptiblue reagent (*n* = 6). We verified that >90% cells were successfully transduced by each of the retroviral vectors on the basis of coexpression of eGFP from an IRES.
(5.01 MB TIF)

Figure S6 **GATA-1/pRb interaction is necessary for GATA-1–mediated terminal erythroid differentiation of GAK14.** (A) The erythroid cell line GAK14 cells, which is defective in GATA-1 as it derives from GATA-1.05 ES cells (M. Yamamoto, unpublished data) were grown on OP9 stromal cells in the presence of erythropoietin and stem cell factor. GAK14 cells were transduced with retroviral vectors expressing hGATA-1 or hGATA-1Rb^−^. Differentiation of the cells was studied at Day 7 posttransduction by May- Grünwald-Giemsa staining. (B) Retrovirally transduced GAK14 cells were analyzed by microscopy and the distribution of cells at distinct stages of erythroid differentiation scored. More than 300 cells were examined for each sample. Expression of hGATA-1Rb^−^ did not impair the initiation of erythroid differentiation but dramatically alter the distribution of erythroid precursors towards the more immature elements. These results indicate that GATA-1/pRb interaction modulates the proportion of the various erythroid precursors. Baso, basophilic erythroblasts I and II; Ortho, orthochromatophilic erythroblasts; Poly, polychromatophilic erythroblasts; Pro, proerythroblasts.
(3.02 MB TIF)

## Abbreviations

Ab: antibody
co-IP: coimmunoprecipitation
E: embryonic day
HRD: hematopoietic regulatory domain
IP: immunoprecipitation
IRES: internal ribosome entry site
siRNA: small interfering RNA
Tg: transgenic
wt: wild-type
