# Supplementary figures and images for: Direct Binding of pRb/E2F-2 to GATA-1 Regulates Maturation and Terminal Cell Division during Erythropoiesis
Source: PLoS Biol. 2009 Jun 9;7(6):e1000123. doi: 10.1371/journal.pbio.1000123 (PMC2684697; doi:10.1371/journal.pbio.1000123)

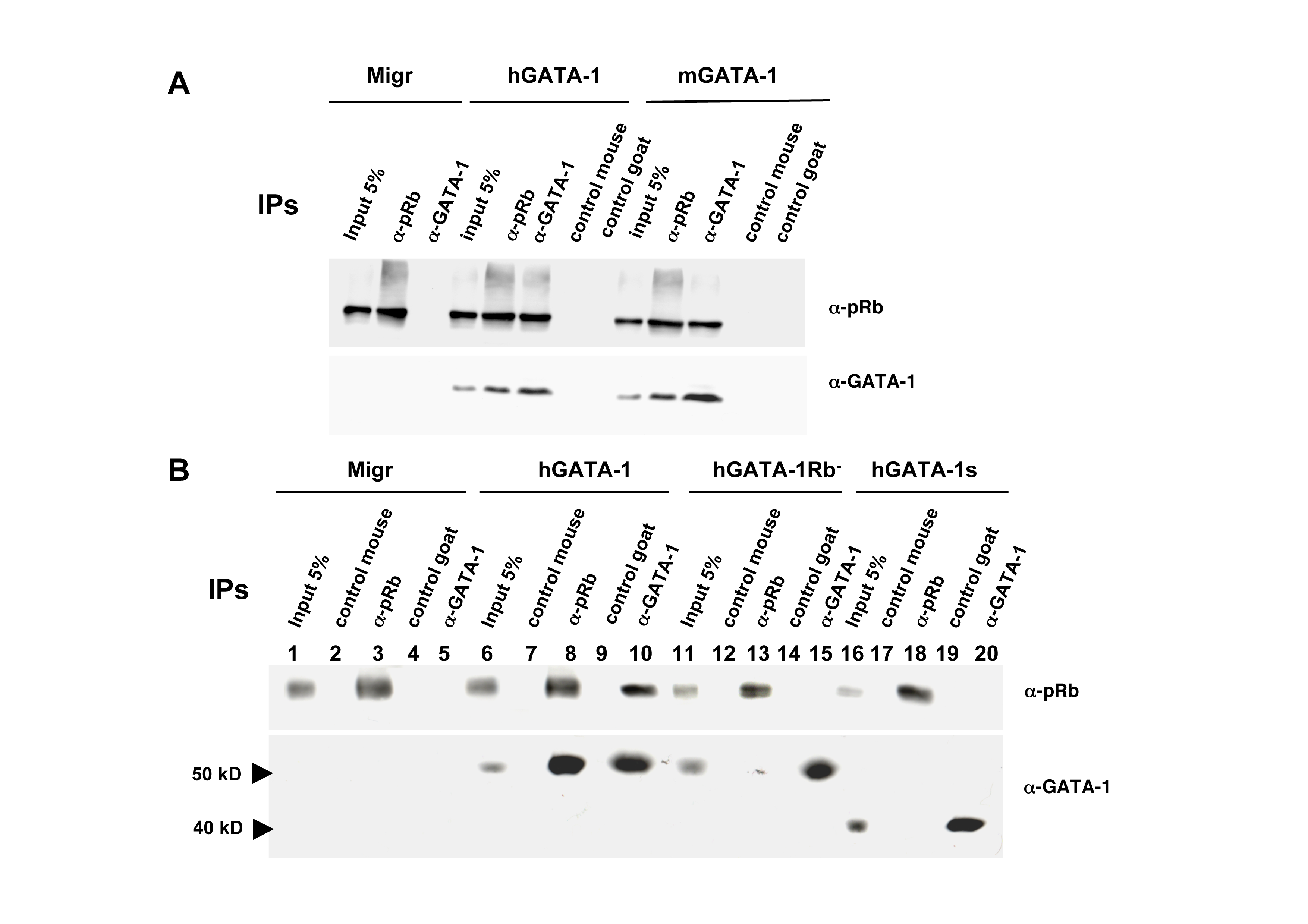

Supplement: Figure S1 — GATA-1 interaction with endogenous pRb and antibody controls. (A) Human and murine GATA-1 proteins interact with endogenous murine pRb. NIH-3T3 cells were transduced with the “empty” retroviral vector (Migr) or with retroviral vectors encoding either human (h) or murine (m) GATA-1. We verified that >90% cells were successfully transduced by each of the retroviral vectors on the basis of coexpression of eGFP from an internal ribosome entry site (IRES). IPs of GATA-1 (5 µg, α-GATA-1: M-20, sc-1234; Santa Cruz Biotechnology) or pRb (5 µg, α-pRb, n° 554136; BD Pharmingen) were performed with 500 µg of nuclear extract. IPs using 5 µg of nonimmune IgG isotype of the corresponding species (normal goat IgG sc-2028 for GATA-1 and normal mouse IgG sc-2025 for pRb; Santa Cruz Biotechnology) were used for each transfection as negative controls for the specificity of the precipitation and coprecipitation obtained with each immune antibody used in the IP and co-IP experiments. Nuclear extracts before IP (input 5%) and bound proteins were resolved by western blot analysis using the antibody against GATA-1 (α-GATA-1 M-20, sc-1234; Santa Cruz Biotechnology) or pRb (α-pRb, n° 554136; BD Pharmingen) as indicated. The horseradish peroxydase-conjugated secondary Ab used (Affinipure goat anti-rat IgG light chain specific, 112-035-175; Affinipure goat anti-mouse IgG light chain specific, 115-035-175) was provided from Jackson Immunoresearch and did not recognize reduced denaturated IgG heavy chains that comigrate at 50 kDa near GATA-1 (http://www.jacksonimmuno.com/catalog/CatPages/rblc.asp). These secondary Abs were used in all the blotting assays of this paper. This experiment indicates that both human and murine GATA-1 interact with endogenous murine pRb with the same efficiency. (B) Control of the specificity of the Abs utilized. NIH-3T3 cells were transfected with the “empty” retroviral vector (Migr) or with retroviral vectors encoding for either hGATA-1, hGATA-1Rb−, or hGATA-1s. IP o [file pbio.1000123.s001.tif]

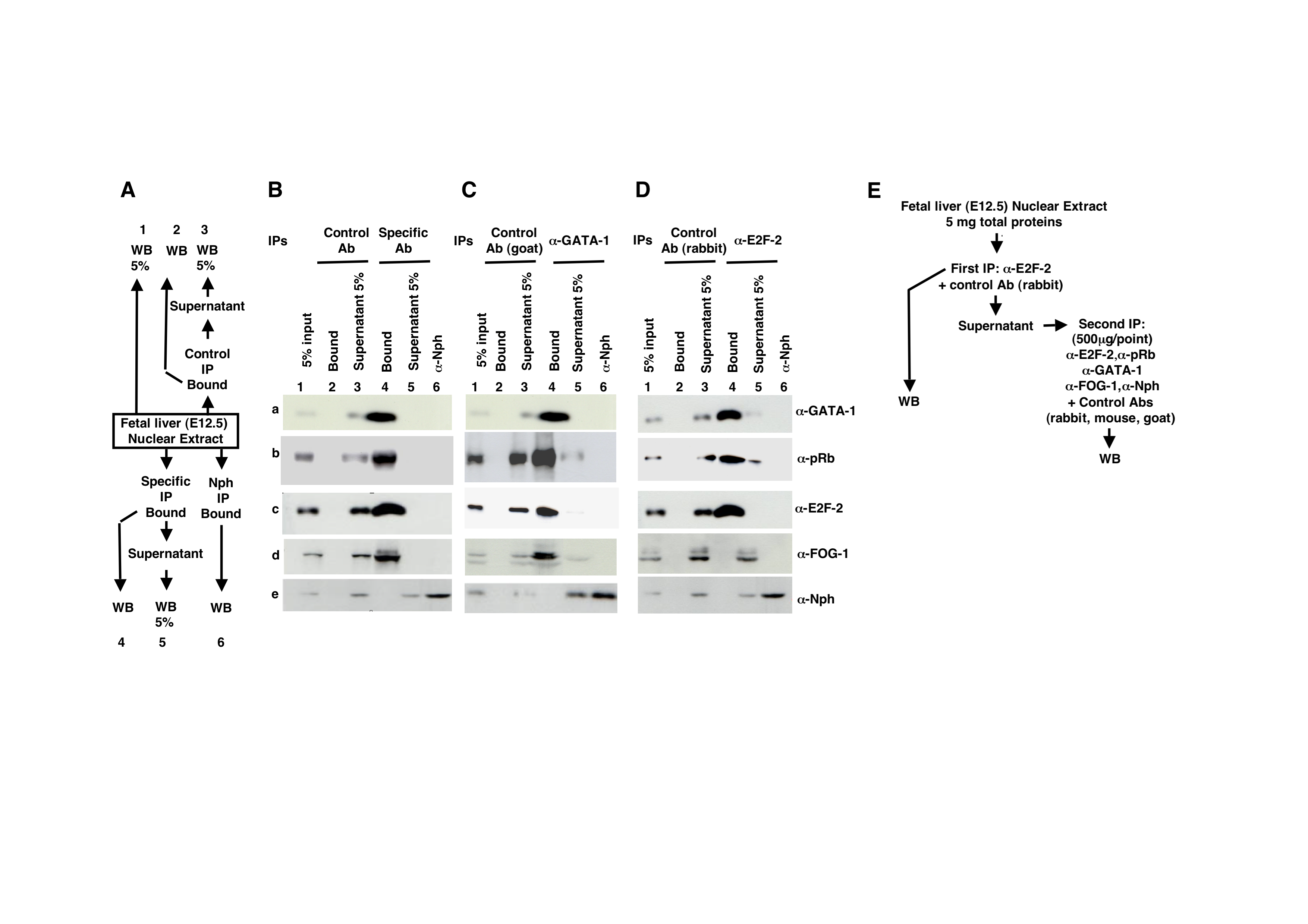

Supplement: Figure S2 — GATA-1 forms a complex with pRb and E2F-2 in primary erythroid cells. (A) Experimental procedures used for (B–D). Fetal livers were dissected from C57BL/6 embryos at embryonic day 12.5 (E12.5) and disaggregated in α-MEM supplemented with phosphotyrosine phosphatase inhibitors (1 mM Na2VO4; Sigma), phosphoserine/threonine phosphatase inhibitors (20 mM NaF, 1 mM sodium pyrophosphate, 25 mM β-glycerophosphate; Sigma), and proteinase inhibitors (Roche) to a single-cell suspension by serial passage through a 23-ga needle, followed by a 27-ga needle. Viability of cells was assessed by Trypan blue staining (<0.5% of positive cells), and erythroid differentiation stages were checked by cell surface labeling using the following Abs: phycoerythrin-conjugated anti-mouse TER119 (TER119-PE; BD Pharmingen, cat N°553673), APC-conjugated anti-mouse CD117 (c-Kit; BD Pharmingen, cat N°553356), biotin-conjugated anti-mouse CD71 (BD Pharmingen, cat N°557416), and streptavidin-PEPC5 secondary antibodies (BD Pharmingen). Flow cytometry was performed on an aliquot of 105 cells using FACScan, and data were analyzed with the DIVA software. Eighty-five percent to 90% of fetal liver cells were CD71+ TER119+ c-Kit−, showing a majority of cells at the late stage of erythroid differentiation. Nuclear extract were prepared as described in Materials and Methods except that the glycerol concentration was 10% instead of 5% to allow for an improved recovery of nuclear pellets. IPs were then performed on equal amounts of nuclear extract (500 µg per point) using specific Abs or nonimmune-related (control) Abs. Immunoprecipitated proteins (bound, lanes 2 and 4) were analyzed by western blot (WB) as well as a 5% fraction of nuclear extract before (input, lane 1) and after (supernatant, lane 3 and 5) each IP. Nucleophosmin (Nph), which does not bind to GATA-1 or pRb complexes, was used as a negative control of the specificity of coprecipitation (lane 6). (B) Efficiency and specificity of immunoprecipitati [file pbio.1000123.s002.tif]

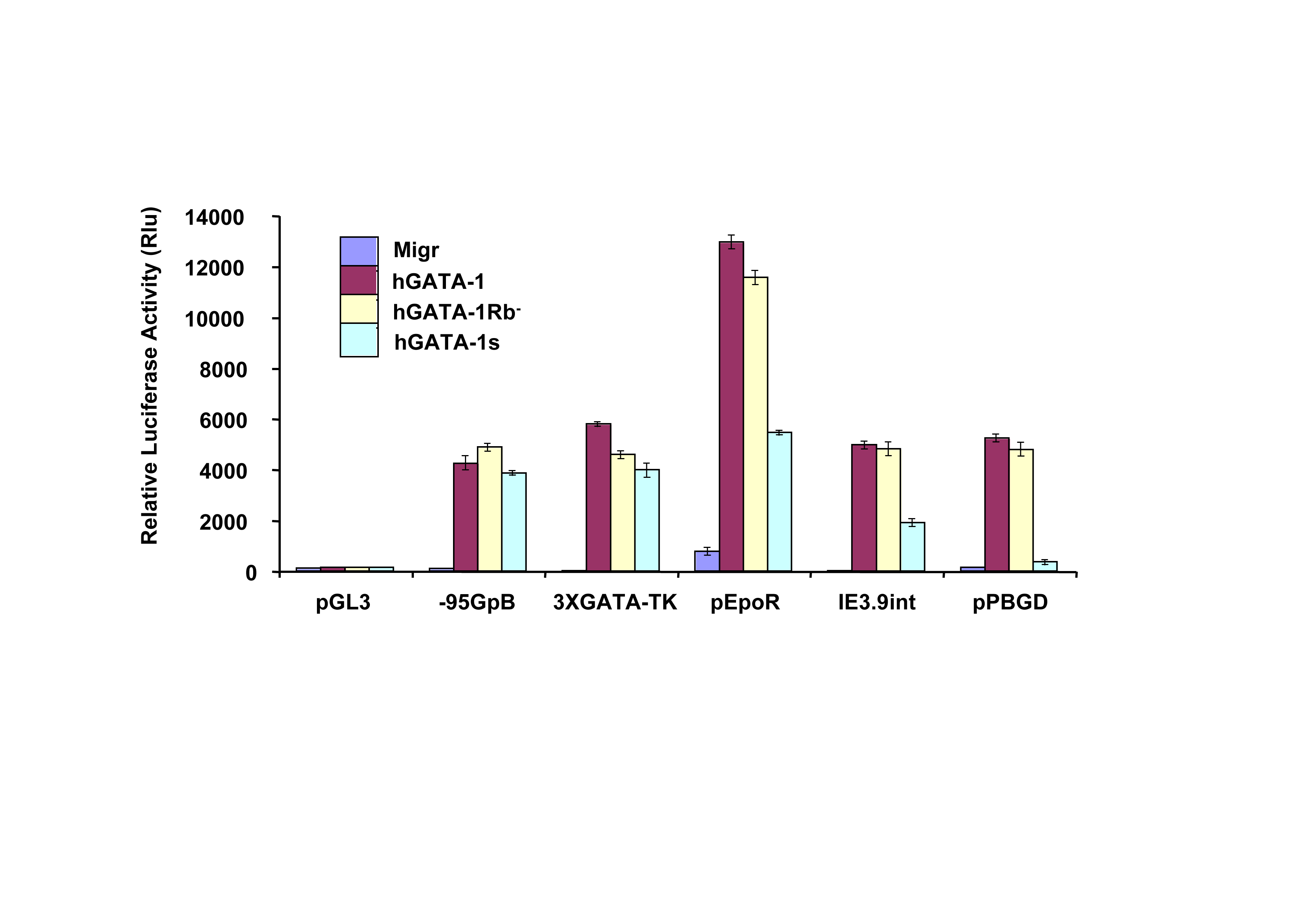

Supplement: Figure S3 — hGATA-1 and hGATA-1Rb − proteins have undistinguishable transcriptional activity. To assess the respective transcriptional activities of hGATA-1, hGATA-1Rb−, and hGATA-1s, we performed transient transfection assays in NIH-3T3 cells using a luciferase reporter gene driven by several minimal erythroid-specific promoters that included (1) the erythroid porphobilinogene deaminase promoter (nucleotides −714 to +78 PBGD) [42], (2) the glycophorin-B promoter (−95GpB) [43], (3) the erythropoietin receptor promoter (−58, +43 Epo-R) [44], (4) the 3XGATA-TK synthetic promoter [45], and (5) the GATA-1 proximal promoter (IE) together with its upstream enhancer element (IE3.9int) [46]. These different plasmids were cotransfected with the “empty” Migr plasmid or with Migr-derived plasmids expressing hGATA-1, hGATA-1Rb−, or hGATA-1s. Firefly luciferase activity was measured according to the manufacturer's instructions (Dual Luciferase Reporter Assay System; Promega), and individual transfections were normalized by quantification of Renilla luciferase activity (pRL-TK; Promega). The total amount of DNA was kept constant at 700 ng in each transfection (500 ng of expression plasmid, 100 ng of reporter plasmid, and 100 ng of pRL-TK plasmid per well of a 24-well plate). Luciferase activity was determined 24 h after transfection. Data are expressed as relative luciferase activity (RLu). Results are the means±standard error of the mean (SEM) of three independent experiments. No difference in the transcriptional activity of hGATA-1 and hGATA-1Rb− was detected with these promoters. With respect to hGATA-1s, the Epo-R minimal erythroid (pEpoR) and the GATA-1 (IE3.9int) promoters were activated at a lower level (approximately 50%), whereas the erythroid porphobilinogene deaminase promoter was not activated, and both the glycophorin-B (−95GpB) and the synthetic 3XGATA-TK promoters were equally transactivated. (1.07 MB TIF) [file pbio.1000123.s003.tif]

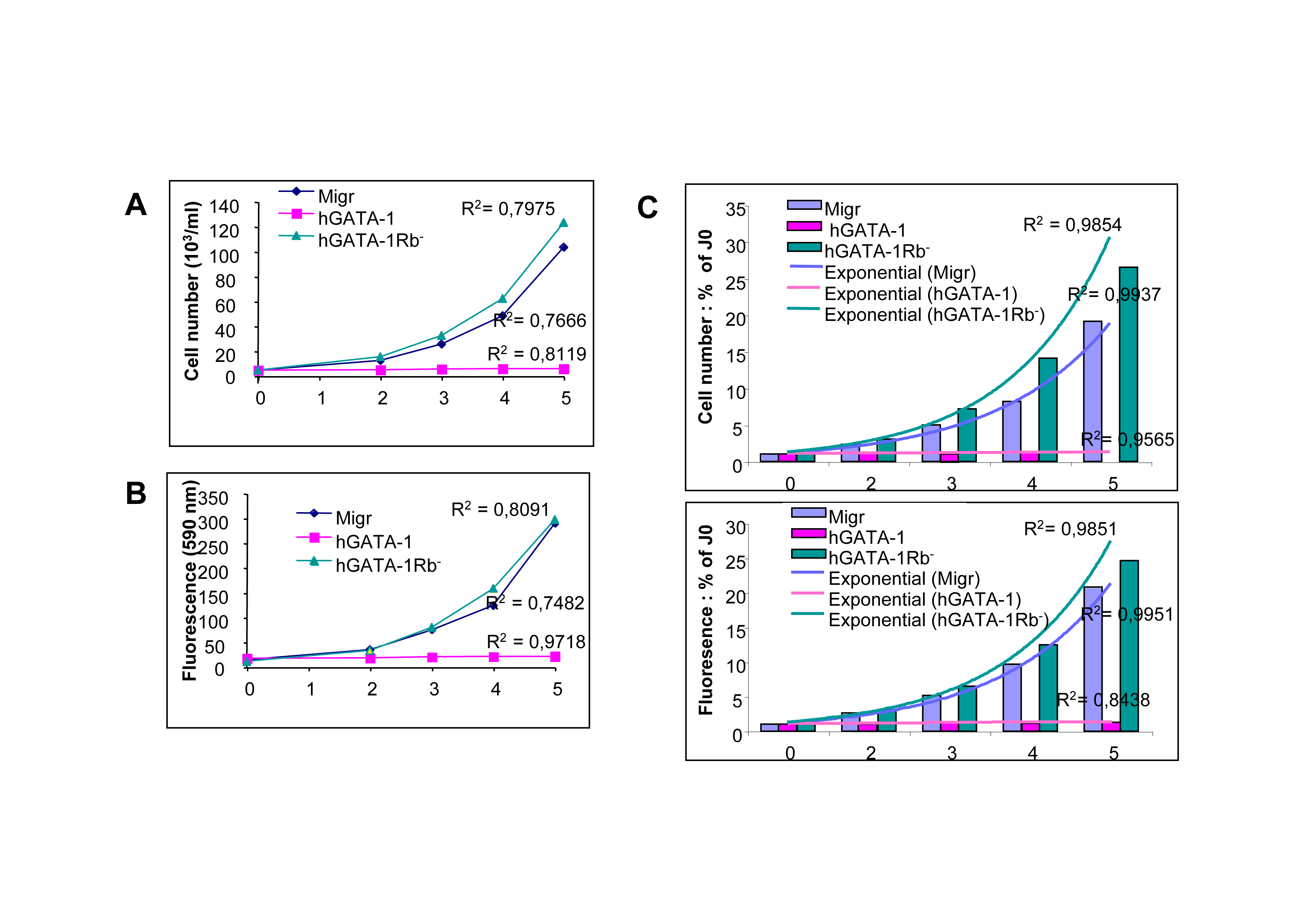

Supplement: Figure S4 — Comparison of the two different methods used to monitor cell proliferation. Retroviral vectors encoding hGATA-1 or the hGATA-1 mutant that cannot interact with pRb (hGATA-1Rb−) were used to transduce NIH-3T3 cells (100% eGFP-positive cells). Proliferation of the transduced NIH-3T3 cells was monitored each day for 5 d. For cell growth analysis, identical numbers of NIH-3T3 cells transduced with retroviral vectors expressing hGATA-1 or hGATA-1Rb− were plated on Day 1. (A) At each indicated day, the number of viable cells was determined by trypan blue dye exclusion (n = 3). Mock indicates the “empty” Migr retroviral transduction. (B) Representative growth curve obtained by using the fluorimetric metabolic growth indicator Uptiblue reagent (n = 3). (C) Comparison of the analysis obtained with the two methods used in (A) and (B): each data point obtained in (A) was converted into a percentage of cell numbers comparatively to the initial number of cells at Day 0 (top). Each data point obtained in (B) was converted into a percentage of the 590-nm fluorescence in arbitrary units comparatively to that measured at Day 0 (bottom). The resulting curves and the R 2 values were determined by the Excel software (Microsoft). The observation that the deduced R 2 curves are of identical value indicates that the two methodologies are comparable. The proliferation of NIH-3T3 cells can be thus monitored by either trypan blue dye exclusion or Uptiblue reagent. (1.78 MB TIF) [file pbio.1000123.s004.tif]

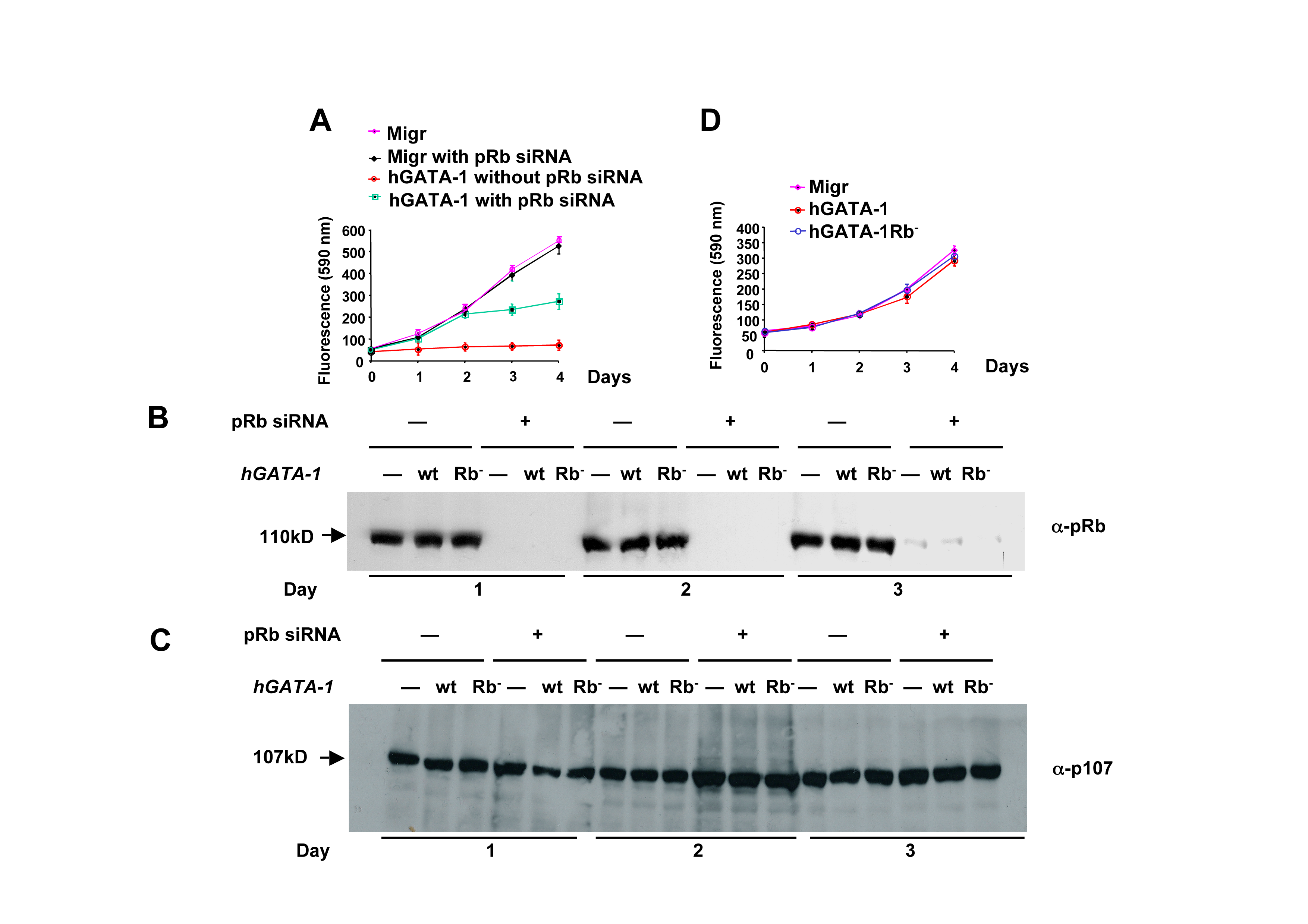

Supplement: Figure S5 — GATA-1/pRb interaction is required for GATA-1–mediated arrest of cell proliferation. (A) Same as in Figure 3A, except that the hGATA-1Rb− retrovirus was not used here and that proliferation was monitored in the presence of a siRNA directed against endogenous pRb. The siRNA was transfected at Day 0 of the experiment (n = 4). (B) Retroviral vectors expressing hGATA-1 or hGATA-1Rb− were used to transduce NIH-3T3 cells in the absence (−) or in the presence (+) of an siRNA directed against endogenous pRb (sc-29469; Santa Cruz Biotechnology). pRb knock-down analysis was performed each day for three consecutive days. Total proteins were subjected to western blot analysis using an anti-pRb antibody (BD Pharmingen). (C) Membranes were then stripped and reprobed with an anti-p107 antibody (sc 318; Santa Cruz Biotechnology). These data indicate that pRb protein levels were very low shortly after transfection of the siRNA directed against pRb and started to increase 3 d thereafter. The level of pRb expression at Day 3 correlates with the decrease in cell proliferation observed the same day with NIH-3T3 cells transduced with a retroviral vector expressing hGATA-1 and transfected with the pRb siRNA [see (A)]. (D) The same retroviral vectors as in (A) were used to transduce the human cell line SAOS-2, which expresses neither GATA-1 nor pRb endogenously. Cell proliferation was monitored each day for 4 d with the Uptiblue reagent (n = 6). We verified that >90% cells were successfully transduced by each of the retroviral vectors on the basis of coexpression of eGFP from an IRES. (5.01 MB TIF) [file pbio.1000123.s005.tif]

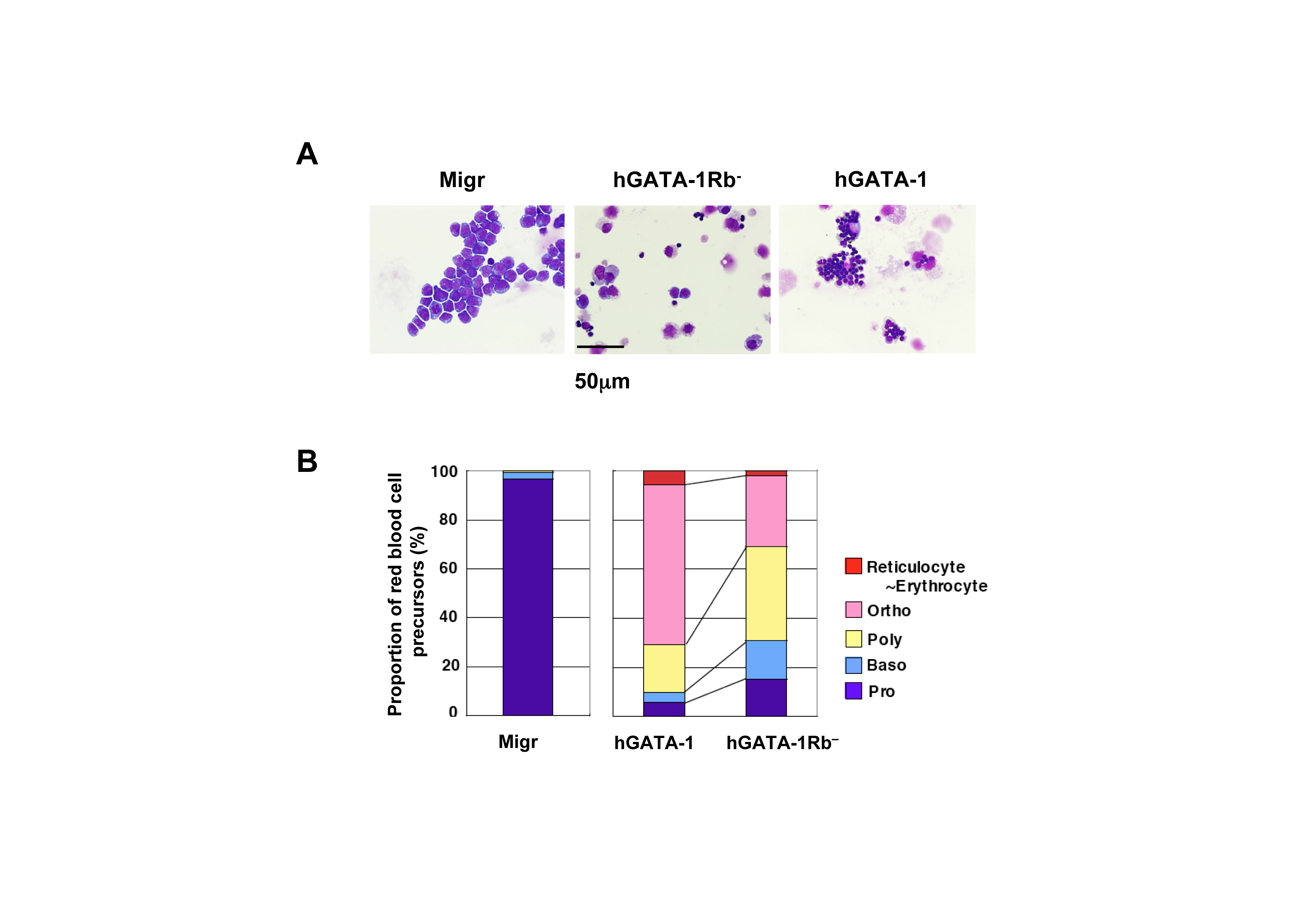

Supplement: Figure S6 — GATA-1/pRb interaction is necessary for GATA-1–mediated terminal erythroid differentiation of GAK14. (A) The erythroid cell line GAK14 cells, which is defective in GATA-1 as it derives from GATA-1.05 ES cells (M. Yamamoto, unpublished data) were grown on OP9 stromal cells in the presence of erythropoietin and stem cell factor. GAK14 cells were transduced with retroviral vectors expressing hGATA-1 or hGATA-1Rb−. Differentiation of the cells was studied at Day 7 posttransduction by May- Grünwald-Giemsa staining. (B) Retrovirally transduced GAK14 cells were analyzed by microscopy and the distribution of cells at distinct stages of erythroid differentiation scored. More than 300 cells were examined for each sample. Expression of hGATA-1Rb− did not impair the initiation of erythroid differentiation but dramatically alter the distribution of erythroid precursors towards the more immature elements. These results indicate that GATA-1/pRb interaction modulates the proportion of the various erythroid precursors. Baso, basophilic erythroblasts I and II; Ortho, orthochromatophilic erythroblasts; Poly, polychromatophilic erythroblasts; Pro, proerythroblasts. (3.02 MB TIF) [file pbio.1000123.s006.tif]
